# Two-dimensional nanomaterials based on rare earth elements for biomedical applications

**DOI:** 10.1039/d4sc02625j

**Published:** 2024-09-19

**Authors:** Mingjun Bai, Hao Wan, Ying Zhang, Siqi Chen, Chunyin Lu, Xiaohe Liu, Gen Chen, Ning Zhang, Renzhi Ma

**Affiliations:** a School of Materials Science and Engineering, Chongqing University of Technology Chongqing 400054 P. R. China; b Zhongyuan Critical Metals Laboratory, Zhengzhou University Zhengzhou 450001 P. R. China wanhao@zzu.edu.cn liuxh@csu.edu.cn; c School of Materials Science and Engineering, Central South University Changsha 410083 P. R. China; d Research Center for Materials Nanoarchitectonics (MANA), National Institute for Materials Science (NIMS) Tsukuba Ibaraki 305-0044 Japan MA.Renzhi@nims.go.jp

## Abstract

As a kind of star materials, two-dimensional (2D) nanomaterials have attracted tremendous attention for their unique structures, excellent performance and wide applications. In recent years, layered rare earth-based or doped nanomaterials have become a new important member of the 2D nanomaterial family and have attracted significant interest, especially layered rare earth hydroxides (LREHs) and layered rare earth-doped perovskites with anion-exchangeability and exfoliative properties. In this review, we systematically summarize the synthesis, exfoliation, fabrication and biomedical applications of 2D rare earth nanomaterials. Upon exfoliation, the LREHs and layered rare earth-doped perovskites can be dimensionally reduced to ultrathin nanosheets which feature high anisotropy and flexibility. Subsequent fabrication, especially superlattice assembly, enables rare earth nanomaterials with diverse compositions and structures, which further optimizes or even creates new properties and thus expands the application fields. The latest progress in biomedical applications of the 2D rare earth-based or doped nanomaterials and composites is also reviewed in detail, especially drug delivery and magnetic resonance imaging (MRI). Moreover, at the end of this review, we provide an outlook on the opportunities and challenges of the 2D rare earth-based or doped nanomaterials. We believe this review will promote increasing interest in 2D rare earth materials and provide more insight into the artificial design of other nanomaterials based on rare earth elements for functional applications.

## Abstract 2

Two-dimensional rare earth-containing nanomaterials are promising candidates in biomedical applications such as drug delivery, bio-imaging, antibacterial and cancer treatment due to their unique structure and versatile energy levels.

## Graphical abstract

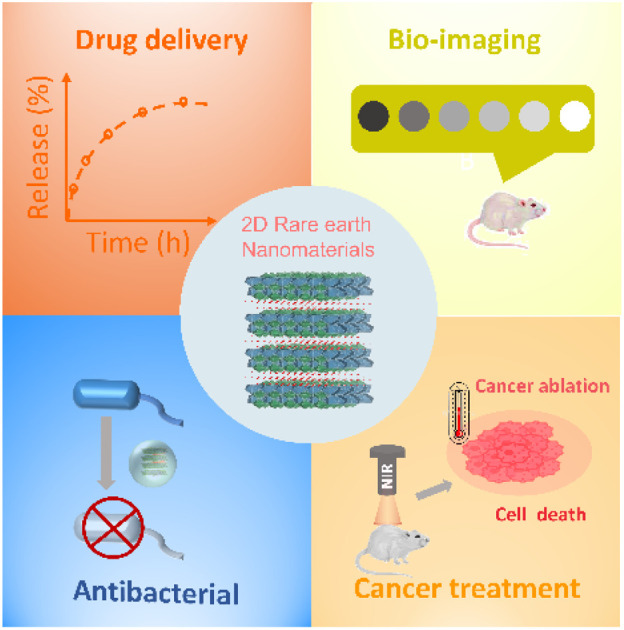

## 1. Introduction

Two-dimensional (2D) materials are defined as crystalline materials of single- or few-layer atoms or molecules, in which the in-plane interatomic interactions are much stronger than those along the stacking direction.^1^ After the exfoliation of layered graphite into graphene, the 2D materials have grown to a large family including the graphene family (*e.g.*, graphene, graphyne, hexagonal boron nitride (hBN), black phosphorene (BP)),^2–6^ Xenes (*e.g.*, borophene, phosphorene, silicene),^7–10^ chalcogenides (*e.g.*, MoS_2_, MoSe_2_, Sb_2_Te_3_),^11–13^ MXenes (*e.g.*, Ti_3_C_2_, Nb_2_C, Ta_4_C_3_),^14–16^ metal–organic frameworks (MOFs),^17–19^ covalent organic frameworks (COFs),^19–21^ 2D oxides (*e.g.*, 2D semiconductor oxides, 2D perovskites),^22–26^ 2D hydroxides (*e.g.*, layered double hydroxides (LDHs), and layered rare earth hydroxides (LREHs)).^2,27–33^ The explosive growth of the 2D family offers a huge opportunity for the design of crystal structures. The rare earth-based or doped nanomaterials contain a large collection. This review mainly focuses on LREHs, layered rare earth-containing perovskites and their exfoliated ultrathin nanosheets due to their unique structures and fascinating physicochemical properties.

Rare earth-based or doped 2D nanomaterials with versatile energy levels of rare earth/lanthanide (Ln) ions and various active sites are widely applied in diverse fields such as luminescence, sensors, catalysis, *etc.*^34–39^ Due to the spatial localization of 4f electrons within the RE atoms, which induces an unquenched total angular momentum, rare earth-containing nanomaterials exhibit excellent magnetic properties. Among them, rare earth-transition metal nanostructures such as SmCo_5_ and Nd_2_Fe_14_B are promising permanent magnets.^40^ In recent years, ultrathin 2D materials (*e.g.*, graphene, MXene, BP, *etc.*) have been reported showing great potential in biomedical fields. For 2D rare earth compounds, in addition to luminescence properties, more and more research studies have been devoted to the biomedical field such as magnetic resonance imaging (MRI), drug delivery, photothermal therapy (PTT) and photodynamic therapy (PDT), especially for Gd^3+^-containing materials, due to the half-filled 4f electron structure, good flexibility and biocompatibility.^41–47^

Similar to LDHs, LREHs are host–guest compounds with rare earth ions occupying the host layers while anions are in the interlayer gallery. After the first preparation in the 1960s, the LREH family expanded to accommodate nearly all rare earth elements from La to Tm and Y and Lu. Various rare earth elements endow LREHs with color-changeable luminescence.^32,34,37,48^ Recently, *in vivo* upconversion imaging and MRI performances based on upconversion luminescence and magnetic properties have received much attention, especially for Gd^3+^, Yb^3+^ and Nd^3+^ ion-containing host layer LREHs.^45,49–53^ Extensive research has proved that LDHs are good carriers of multiple biomedical active species such as deoxyribonucleic acid (DNA), peptides, proteins, vitamins, *etc.*^54–56^ With similar layered structures, LREHs and other layered rare earth materials are also promising carriers of diverse biomedical drugs, for example, aspirin.^44,57,58^ Moreover, combined with the special luminescence and magnetic properties of RE^3+^, multifunctional drug delivery methods can be realized on the rare earth-containing layered materials, such as targeted drug delivery and real-time monitoring of drug release conditions. Compared with non-layered materials, the interlayer space and rich ion exchangeability of layered rare earth-containing nanomaterials are beneficial to promoting drug loading and release. After exfoliation, the ultra-thin nanosheets expose multiple active sites, ensuring further modification and assembly to facilitate biomedical applications. In addition, unlike common 3D bulk materials, ultra-thin nanosheets are less likely to settle in biological tissues and therefore exhibit higher biocompatibility. The unique layered structure of LREHs enables exfoliation into single or several layers. The ultrathin nanosheets obtained are desirable building blocks to construct artificial superlattice structures due to their large lateral size-to-thickness ratio and high flexibility.^34,59–61^ Furthermore, the hydrophilicity and biocompatibility of the hydroxide nanosheets make them a promising contrast agent for MRI toward tumor vaccination.^42,43,62^

Though the LREH nanosheets show huge application potential, hydroxyl groups and absorbed water molecules in the rare earth hydroxide nanosheets weaken the photoluminescence properties. Also, the inferior stability of hydroxide nanosheets limits their applications. Therefore, considering the stability, composition and structure variability of nanosheets, more efforts are needed to widen the application potential of rare earth nanosheets.

In addition to LREHs, layered rare earth doped perovskites are another class of 2D rare earth-containing nanomaterials. The diverse compositions and structures of perovskites endow them with various properties and wide applications and have become a target topic in recent years. According to the anion types, the perovskite materials can be divided into two types, namely halide perovskites and oxides. Compared with bulk perovskite, the perovskite nanosheets present a series of advantages, such as higher processability, which is more conducive to the manufacturing of thin films and flexible devices; more active sites, which is more conducive for catalysis; and small size effect and quantum effect, which are better for optical, electrical, and magnetic properties.^38,63,64^ Soft chemistry methods are commonly used for the exfoliation of layered perovskites, that is, dispersing the pretreated layered rare earth perovskite into an organic solution with continuous agitation or ultrasonication. Through this method, diverse perovskite nanosheets were prepared, such as La_0.90_Eu_0.05_Nb_2_O_7_, Eu_0.56_Ta_2_O_7_, La_1−*x*_Tb_*x*_Ta_2_O_7_, La_0.95_Eu_0.05_Nb_2_O_7_ single perovskite nanosheets and (K_1.5_Eu_0.5_)Ta_3_O_10_, Gd_2−*x*_Eu_*x*_Ti_3_O_10_, GdMgWO_6_:Eu double perovskite nanosheets. Atomic force microscopy (AFM) results indicate a single-layer structure with lateral size up to 4 mm.^65–69^ Moreover, the layered rare earth-containing perovskite crystal or nanosheet can be applied in photoluminescence, antibacterial activity studies, photocatalysis and bioimaging.^70–72^

**Fig. 1:**
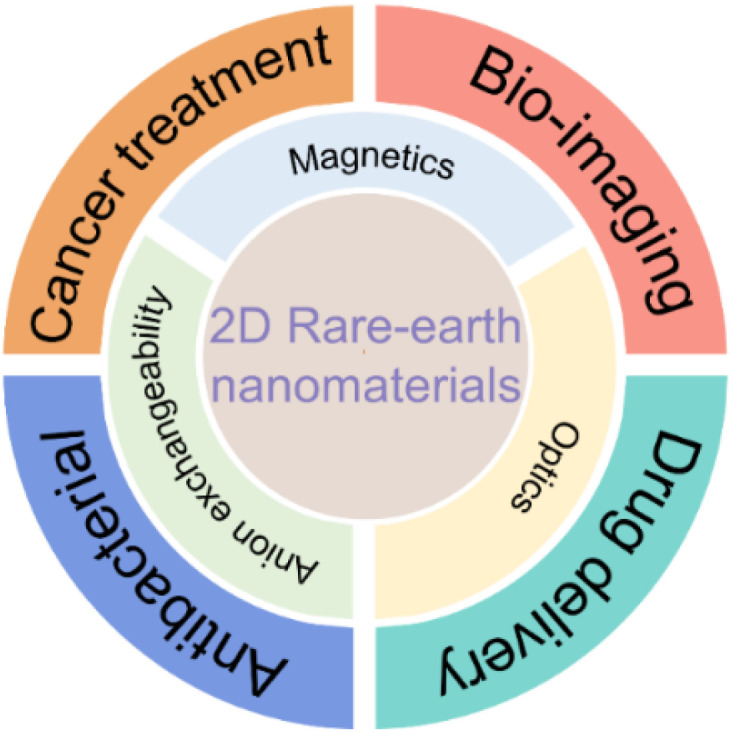
Schematic illustration of the properties and biomedical applications of rare earth nanomaterials.

Therefore, with the combined advantages of flexible sheet-like morphology and rich rare earth composition, nanosheets based on rare earth elements and their assemblies are promising materials in many fields, especially in biomedical applications (Fig. 1). To date, there are some excellent reviews on LREHs and/or rare earth-doped oxides focusing on their preparation, crystal structure, exfoliation and photoluminescence properties.^31,32,73–75^ On the other hand, for 2D materials in biomedical applications and/or biosensors, the published reviews mainly focus on C_3_N_4_, BP, transition metal dichalcogenides (TMDs) and MXene.^76,77^ Few of them have paid sufficient attention to the biomedical applications of 2D rare earth-based or doped nanomaterials. Thus, considering the great potential of emerging 2D rare earth-based or doped nanomaterials, we review the preparation, structural evolution and biomedical applications of 2D nanomaterials based on rare earth elements. The challenges and future perspectives of utilizing these emerging 2D nanomaterials in biomedical fields are also provided.

## 2. Preparation and structural evolution of 2D rare earth compounds

Various methods have been developed to prepare 2D materials and are mainly divided into two strategies, *i.e.*, “bottom-up” and “top-down” approaches. Molecular beam epitaxy (MBE), chemical vapour deposition (CVD) and pulsed laser deposition (PLD) are typical bottom-up strategies.^78^ Diverse 2D rare earth materials such as Yb-doped WS_2_, EuC_6_, GdSi_2_ and WS_2_(Er^3+^)/WSe_2_(Er^3+^) were prepared through this bottom-up method, holding potential for uses in next-generation optoelectronic devices.^79–82^ But silicon or sapphire wafers are needed for the bottom-up processes, limiting the application in biomedical fields. On the other hand, mechanical exfoliation and soft-chemical exfoliation are well-known top-down methods and widely used in the preparation of ultra-thin rare earth-containing 2D nanosheets, especially for LREHs and rare earth-containing perovskites.^25,33,65,66,68^

### 2.1 Preparation and exfoliation of LREHs

**Fig. 2:**
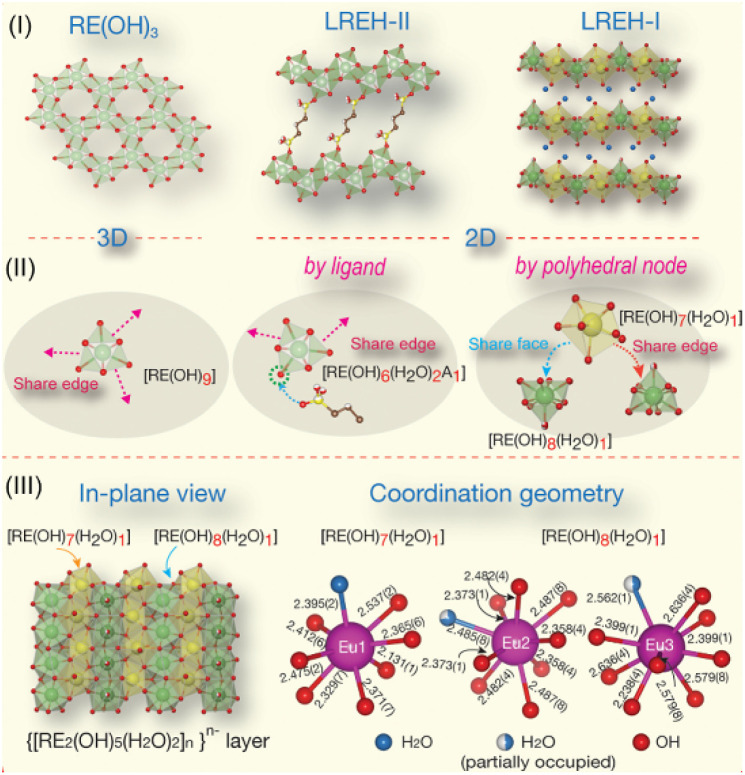
Structure illustrations of LREHs. (I) RE(OH)_3_, LREH-II and LREH-I structures. (II) Connectivity of the polyhedral unit for the three structures. (III) In-plane view of the anion-exchangeable LREH-I structure and representative coordination geometries of RE cations. Adapted from ref. 31. Copyright 2023, Elsevier Inc.

LREHs with a typical formula of RE_2_(OH)_6−*m*_[A^*x*−^]·H_2_O, in which A^*x*−^ represents intercalated anions such as Cl^−^ or CO_3_^2−^, have emerged as one of the most important 2D rare earth compounds since their first proposal in 2006.^83^ Similar to LDHs, the LREHs are composed of positively charged rare earth hydroxide layers and negatively charged interlayer anions. The host layers and guest anions are linked through strong chemical bonds between the RE^3+^ cations and interlayer anions, resulting in anion-exchangeable properties.^83–87^ According to the coordination situation, the LREHs can be divided into two types, namely LREH-I and LREH-II. For the former, one RE^3+^ is coordinated by seven OH^−^ groups and one H_2_O molecule, while for the later one, each RE^3+^ is coordinated by six OH^−^ groups, two H_2_O molecules and one anionic ligand, as shown in Fig. 2.^31,88^ In addition, for LREH-I, the interlayer anions, screened from the direct coordination of RE^3+^ by H_2_O molecules, result in a weaker bond energy and thus a more easier interlayer anion-exchange process.^31^

**Fig. 3:**
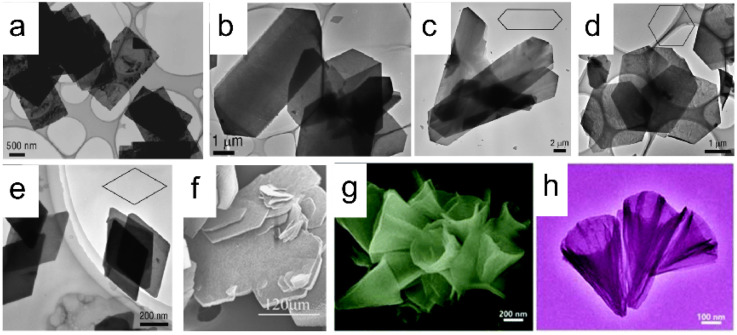
Scanning electron microscopy (SEM) and transmission electron microscopy (TEM) images of synthesized LREHs. (a) As-prepared LEuH through the homogeneous precipitation method. Adapted from ref. 88. Copyright 2008, John Wiley & Sons, Inc. (b) As-prepared LYH through the homogeneous precipitation method. Adapted from ref. 85. Copyright 2008, American Chemical Society. As-prepared (c) LGdH, (d) LDyH, (e) LErH through the homogeneous precipitation method. Adapted from ref. 89. Copyright 2009, American Chemical Society. (f) As-prepared LYH through the hydrothermal method with NH_4_NO_3_ as a mineralizer. Adapted from ref. 90. Copyright 2012, Elsevier Inc. (g and h) As-prepared LYH through the hydrothermal method with SDS as a surfactant. Adapted from ref. 48. Copyright 2017, the Royal Society of Chemistry.

The commonly used method for the synthesis of LREHs is the co-precipitation method. Through this method, researchers have synthesized diverse LREHs which contain almost all RE elements. With the modification of using hexamethylenetetramine (HMT) or ammonia as the alkali source, Geng *et al.* prepared a series of well-crystallized LREHs with Cl^−^ or NO_3_^−^ intercalation, and systematically analyzed the morphological and crystal structure (Fig. 3a–e).^85,88,89,91,92^ Through direct synthesis and further ion-exchange processes, researchers expanded the LREHs to Br^−^ and I^−^ intercalated counterparts.^86,93^ Though the morphology and crystal structure of LREHs are fully analyzed, the crystal size of the synthesized LREHs is small which is adverse to the following exfoliation process for the preparation of monolayers or few-layer nanosheets. In 2012, Li *et al.* further improved the synthesis process by introducing NH_4_NO_3_ as a mineralizer, which expanded the LREH crystal up to 300 mm (Fig. 3f).^90,94^ Using sodium dodecyl sulfate (SDS) as a surfactant, Zhong *et al.* prepared DS^−^-intercalated LREHs (RE = Y, Tb, Er) with a nanocone morphology (Fig. 3g and h).^49,95^ Therefore, various LREHs were synthesized with diverse morphologies.

As one of the most important features, LREHs exhibit anion-exchange properties. Through uniformly dispersing the as-prepared LREHs with target anions in deionized water under continuous stirring or shaking, LREHs can be topotactically changed to target anion-intercalated counterparts, expanding the LREH family since some functional anionic species are unlikely to intercalate into the interlayer through direct synthetic strategies. There are various interlayer anionic species, including negatively charged inorganic anions (*e.g.*, PO_4_^3−^, CO_3_^2−^, HPO_4_^2−^, ClO_4_^−^, *etc.*), negatively charged organic anions (*e.g.*, oleic acid, phthalic acid, terephthalic acid, *etc.*), and electrically neutral molecules or polymers (*e.g.*, diclofenac, ibuprofen, naproxen, *etc.*).^57,86,88,93,96–105^ Through anion exchange, the interlayer distance can be adjusted and varies from 0.8 nm to 5.2 nm depending on the anion species. In addition, the anion-exchange property endows LREHs with potential drug delivery properties.^46,57,58,106–108^

**Fig. 4:**
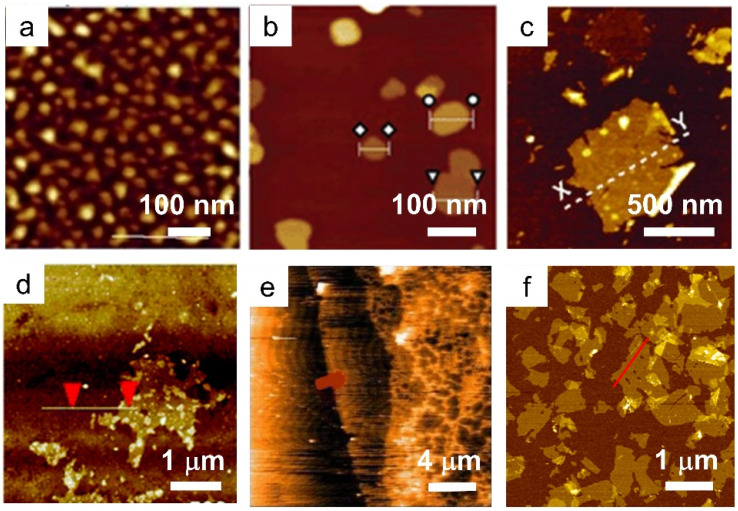
AFM images of LREH nanosheets. (a) LNdH nanosheet through ultrasonication of the NO_3_^−^ intercalated LNdH powder in deionized water. Adapted from ref. 98. Copyright 2009, John Wiley & Sons, Inc. (b) LGdH nanosheet through ultrasonication of the Cl^−^ intercalated LGdH powder in deionized water. Adapted from ref. 41. Copyright 2009, the Royal Society of Chemistry. (c) LEuH nanosheet through shaking of the DS^−^ intercalated LEuH powder in formamide. Adapted from ref. 109. Copyright 2010, John Wiley & Sons, Inc. (d) LTmH nanosheet through magnetic stirring of the DS^−^ intercalated LTmH powder in formamide. Adapted from ref. 110. Copyright 2017, the Royal Society of Chemistry. (e) LYH nanosheet through magnetic stirring of the anion-exchanged NO_3_^−^ intercalated LYH powder in toluene. Adapted from ref. 94. Copyright 2015, Springer Nature. (f) LGdH nanosheet through ultrasonication of the DS^−^ intercalated LGdH powder in formamide. Adapted from ref. 61. Copyright 2021, the Royal Society of Chemistry.

Similar to LDHs, the LREHs can also be exfoliated into nanosheets. In 2009, Lee *et al.* delaminated the as-prepared Cl^−^ intercalated layered Gd hydroxide (LGdH-Cl), NO_3_^−^ intercalated layered Nd hydroxide (LNdH-NO_3_) and NO_3_^−^ intercalated layered La hydroxide (LLaH-NO_3_) crystals into corresponding nanosheets in deionized water under sonication conditions (Fig. 4a and b).^41,98^ Transmission electron microscopy (TEM) and AFM analysis results show that the lateral size of the obtained nanosheets is 50–200 nm with a thickness of 2–8 nm. The large thickness indicates that the deionized water may not be a suitable medium for exfoliating the LREHs. Considering the outstanding swelling effect of formamide on LDHs and the similar structure of LREHs to that of LDHs, researchers turned their eyes to formamide. Lee *et al.* dispersed the LGdH-NO_3_ crystal in formamide. After 4 days of stirring, a transparent suspension was obtained. AFM results show that the lateral size of the obtained nanosheet is 30–100 nm and the thickness is dominated by two different values (*i.e.*, 0.7 nm and 1.4 nm).^111^ Although the obtained nanosheets are thinner when exfoliated in formamide, they are not unilamellar (*e.g.*, the crystallographic thickness of 0.65 nm). This may be because of the strong binding force between the host layers and interlayer anions, thus the formamide molecule is hard to intercalate into the guest gallery, resulting in inefficient exfoliation. Moreover, the size of the nanosheets is too small for further assembly and application. To obtain nanosheets of better quality, DS^−^ anions were introduced into the interlayer gallery of LEuH through an anion-exchange process, and shaken in formamide for 2 days at a speed of 170 rpm. After filtration, nanosheets with a lateral size of 500 nm and thickness of 1.6 nm were obtained (Fig. 4c).^109^ Through a similar two-step exfoliation method, namely, the combination of anion exchange and exfoliation, Li *et al.* obtained LTmH, LYbH and LYH nanosheets with a thickness of around 2 nm and a lateral size up to 1.5 μm (Fig. 4d).^105,110^ Moreover, toluene is also used for the exfoliation of oleic acid-intercalated LYH. The as-obtained nanosheets possess a thickness of 1.55 nm with an ultra-large size of up to 20 mm. However, it is difficult to clearly recognize a full nanosheet by either TEM or AFM observations (Fig. 4e).^94^ In addition to the anion exchange method, Yapryntsev *et al.* tried to exfoliate LYH by a rapid expansion of supercritical suspensions, *i.e.*, treating the LYH crystals with supercritical CO_2_ resulted in a drastically increased distance. After a following ultrasonication treatment in toluene solvent, a clear suspension was obtained.^112^ However, the authors did not present any TEM or AFM images to confirm the nanosheets. Considering the excellent exfoliation performance and degradation effect of formamide on DS^−^ intercalated LDHs, Bai *et al.* modified the two-step exfoliation method and proposed a one-step exfoliation method, *i.e.*, directly exfoliating the as-synthesized DS^−^ anion intercalated LREHs in formamide under ultrasonication conditions without any anion-exchange processes.^33,113^ This one-step method avoids the possible breakage of the LREH crystals during the long-time anion exchange process. Furthermore, the ultrasonic treatment substantially reduces the exfoliation time within 1 hour, greatly shortening the reaction time with formamide and avoiding degradation. Through this one-step exfoliation method, a series of LREH nanosheets were prepared with a lateral size of up to 1 mm while the thickness was only 1 nm, indicating a monolayer feature (Fig. 4f).^33,61^ This versatile approach achieves the preparation of relatively large LREH monolayers, but also offers great potential for the subsequent construction of various 2D assemblies with rich functionalities.

Although the ion exchange and exfoliation route provides more possibilities for the synthesis of diverse LREHs and nanosheets, there are some disadvantages. For the ion exchange process, it is hard to completely substitute the original intercalated ions with the targeted one, which may affect its properties. Moreover, it usually needs a long time (7 days or even longer) and continuous shaking or sonication to fulfil ion exchange and exfoliation. The strong shear force or ultrasonic waves cause the phase and structure damage of layered materials, resulting in reduced size which further affects the exfoliation effect.^109^ As for exfoliation, DS^−^ anion intercalation and using formamide as the exfoliation solvent have been proven to be an effective method for the preparation of single-layer LREH nanosheets. But the LREHs undergo degradation or even dissolution in formamide for a long time, especially under shear force/ultrasonic waves. As a result, it is hard to obtain large-sized nanosheets. Moreover, the exfoliation yield is still relatively low.^33,109^ In addition, during the exfoliation, there may be some defects such as oxygen vacancy or metal ion vacancy and/or dangling bonds which may affect the energy level.^61^ However, a systematic study on these aspects is still lacking.

**Fig. 5:**
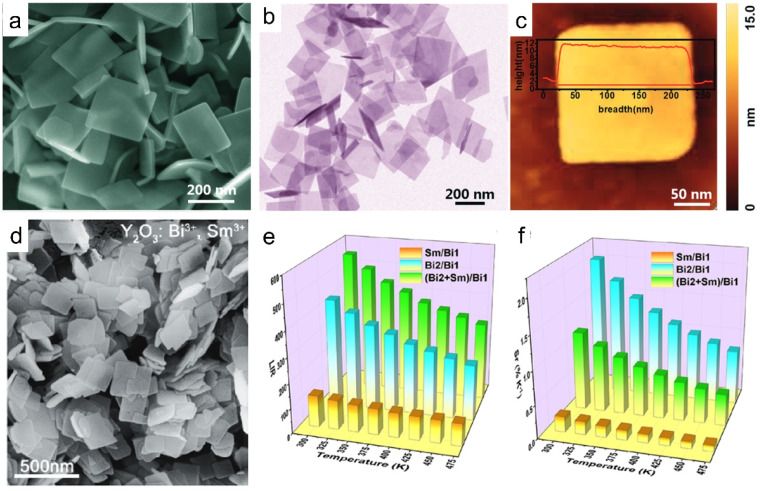
Morphologies of ultrathin rare earth hydroxide or oxide platelets. (a) SEM, (b) TEM and (c) AFM images of Y/Yb/Er hydroxides. Adapted from ref. 50. Copyright 2018, the Royal Society of Chemistry. (d) SEM image, (e) luminescence intensity ratio (LIR) values and (f) relative sensitivity (Sr) values of each mode of the Y_2_O_3_:Bi^3+^,Sm^3+^. Adapted from ref. 114. Copyright 2021, American Chemical Society.

In addition to exfoliation, ultrathin rare earth hydroxides can be prepared by direct synthesis. Through the hydrothermal method with triethylamine (TEA) as a hydrolysis reagent, Xiao *et al.* prepared a series of Y/Yb/Er-hydroxides in the form of nanoplatelets with a thickness of 10 nm, the hydroxides exhibited obvious upconversion properties (Fig. 5a–c).^50^ While co-doping Bi^3+^ and Sm^3+^ into the Y-hydroxides followed by the calcination process, Y_2_O_3_:Bi^3+^,Sm^3+^ tetragonal nanoplatelets were obtained. As the emission peaks present different changing trends with the temperature changes, the Y_2_O_3_:Bi^3+^,Sm^3+^ composite exhibits three kinds of luminescence intensity ratio mode, thus making it a promising temperature sensor with high accuracy (Fig. 5d–f).^114^ Therefore, various kinds of 2D rare earth hydroxides with diverse compositions have been reported till now, which promote their applications in diverse fields.

### 2.2 Preparation and exfoliation of layered rare earth-doped perovskites

Perovskites are a large family with various formulae and structures. Layered perovskites are a sub-set with the general composition of A_*n*−1_B_*n*_X_(3*n*+1)_, in which negatively charged host layers and counter cations in the interlayer space demonstrate some peculiar properties like photocatalytic, conductivity, electronic, and biochemical ones.^72,115–119^ Layered rare earth-doped perovskites are commonly classified into two types, *i.e.*, Dion–Jacobson (DJ) type and Ruddlesden–Popper (RP) type with the general formula of X[A_*n*−1_B_*n*_O_(3*n*+1)_] or X_2_[A_*n*−1_B_*n*_O_(3*n*+1)_], respectively, where X and A are alkali or alkali earth elements, and B represents transition metals such as Ti^4+^, Nb^5+^ or Ta^5+^; rare earth elements usually occupy the A site.^72,117,118^

Layered rare earth-doped perovskites with various compositions are mainly prepared through the solid-state reaction method.^65,67,120^ The exfoliation of layered rare earth-doped perovskites is also a two-step process including ion exchange and exfoliation. Typically, through a replacement of the interlayer alkali metal ion (*e.g.*, K^+^ or Rb^+^) with H_3_O^+^ in acid (*e.g.*, HNO_3_ or HCl) and the swelling process of perovskite bulk in aqueous TBAOH or TMAOH solution with the help of shaking, the nanosheets are obtained.^65,121^

**Fig. 6:**
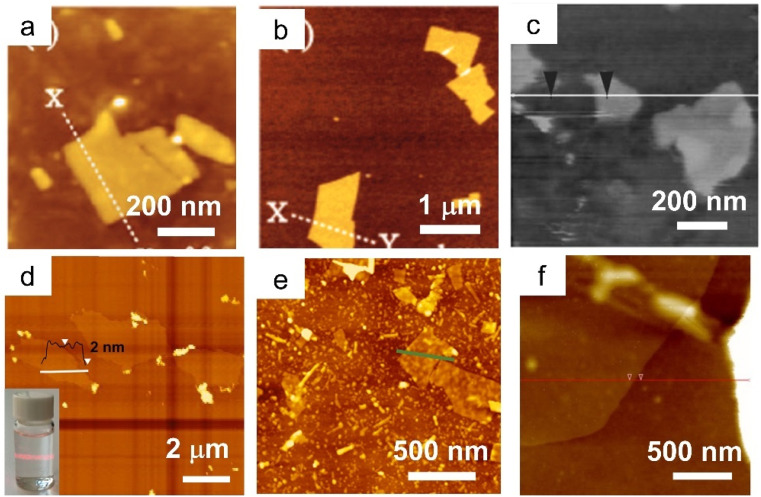
AFM images of rare earth-doped perovskite nanosheets. (a) La_0.90_Eu_0.05_Nb_2_O_7_ nanosheets. Adapted from ref. 67. Copyright 2007, American Chemical Society. (b) Eu_0.56_Ta_2_O_7_ nanosheets. Adapted from ref. 66. Copyright 2008, American Chemical Society. (c) Gd_1.4_Eu_0.6_Ti_3_O_10_ nanosheets. Adapted from ref. 65. Copyright 2008, American Chemical Society. (d) Ln_2_Ti_3_O_10_:Tm^3+^/Er^3+^ nanosheets. Adapted from ref. 122. Copyright 2021, Elsevier Inc. (e) [K_1.5_(Tb_0.8_Sm_0.2_)]Ta_3_O_10_ nanosheets. Adapted from ref. 25. Copyright 2022, the Royal Society of Chemistry. (f) GdMgWO_6_:60%Eu^3+^ nanosheets. Adapted from ref. 69. Copyright 2019, Elsevier Inc.

Based on the successful preparation of LaNb_2_O_7_ nanosheets, Ozawa *et al.* first synthesized a Eu^3+^-doped layered perovskite. After ion exchange and soft chemical exfoliation, La_0.90_Eu_0.05_Nb_2_O_7_ monolayers with a thickness of 2 nm and a lateral size of 400 nm were finally obtained (Fig. 6a).^67,121^ The photoluminescence analysis shows that there are two types of emissions of Eu^3+^ in the bulk, *i.e.*, the direct excited emission and the host excited emission, whereas only host emission exists for the nanosheets, which can be attributed to the difference of the dimensionality and the confinement of the energy-transfer.^67^ Then, they further studied the role of the Eu^3+^ dopant in the photoluminescence properties of Eu_0.56_Ta_2_O_7_ nanosheets (Fig. 6b), and found that the emission intensity originating from the host excitation is much larger than that of the direct excitation of Eu^3+^.^66^ The stronger emission intensity of La_0.90_Dy_0.05_Nb_2_O_7_ nanosheets than that of the bulk also confirmed that the host excitation is more efficient than the direct excitation.^123^ Ida *et al.* expanded the layered rare earth-doped perovskite nanosheets to Gd_1.4_Eu_0.6_Ti_3_O_10_ (Fig. 6c) and La_0.7_Tb_0.3_Ta_2_O_7_, the nanosheets all have a monolayer feature. Benefiting from the energy transfer within the nanosheets from the Ti–O network to Gd^3+^ and then to Eu^3+^, Gd_1.4_Eu_0.6_Ti_3_O_10_ nanosheets show a much stronger emission than La_0.90_Eu_0.05_Nb_2_O_7_ counterparts.^65^ Moreover, they also found that the emission intensity is related to the excitation light and the direction of the magnetic field. Bulk perovskite HLa_2_Ti_2_TaO_10_ with a similar crystal structure was also prepared and further exfoliated into the corresponding La_2_Ti_2_TaO_10_ nanosheets with an ultra-high conductivity range from 10^−9^ to 10^−5^ S cm^−1^.^116^ Beyond the above down-conversion luminescence nanosheets, up-conversion nanosheets of Yb^3+^/Er^3+^, Yb^3+^/Tm^3+^, and Tm^3+^/Er^3+^ co-doped K_2_Ln_2_Ti_3_O_10_ perovskites were also prepared, as shown in Fig. 6d.^122,124,125^

In addition to the above single-layered perovskite, researchers also successfully prepared rare earth-doped double-layered perovskite nanosheets, *e.g.*, (K_1.5_Eu_0.5_)Ta_3_O_10_, (K_1.5_Tb_0.5_)Ta_3_O_10_, and [K_1.5_(Tb_1−*x*_Sm_*x*_)]Ta_3_O_10_ nanosheets (Fig. 6e).^25,68^ Considering the excellent tuneable excitation wavelength and interesting property of cascade energy transfer of double-layered perovskites, researchers designed Eu^3+^-doped double-layered perovskite GdMgWO_6_:Eu nanosheets through proton exchange and exfoliation in ethylamine solution (Fig. 6f).^69,126^ Compared with the bulk perovskite, the GdMgWO_6_:Eu nanosheets showed high concentration quenching of up to 60% of Eu^3+^ and a high quantum yield of 30%.^69^ In general, compared with the bulk phase, the ultrathin nanosheets exhibit enhanced properties in photoluminescence intensity, conductivity and quenching concentration.

### 2.3 Other rare earth-containing 2D materials

**Fig. 7:**
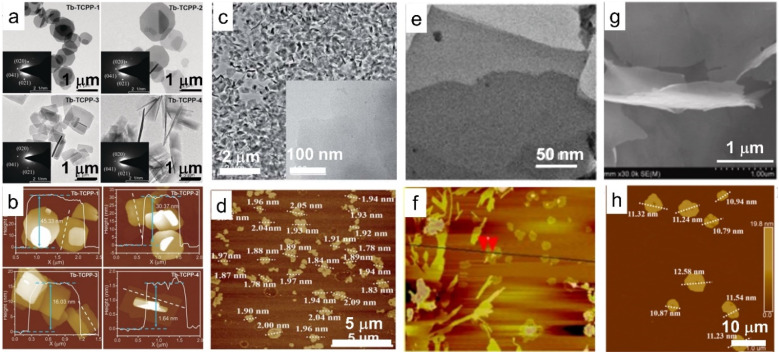
TEM and AFM images of 2D MOF based on rare earth elements. (a) TEM and (b) AFM images of 2D MOFs with Tb as nodes and porphyrin as the ligand (Tb-TCPP). Adapted from ref. 130. Copyright 2020, John Wiley & Sons, Inc. (c) TEM and (d) AFM images of MB/Yb-TCPP-SO_4_, in which MB/Yb-TCPP-SO_4_ represents methylene blue (MB) modified porphyrin-based 2D Yb-TCPP MOF. Adapted from ref. 131. Copyright 2021, the Royal Society of Chemistry. (e) TEM and (f) AFM images of rare earth-containing MOF nanosheets with 2,2′-thiodiacetic acid as a surfactant. Adapted from ref. 132. Copyright 2017, Springer Nature. (g) SEM and (h) AFM images of the 2D Tb/Eu-bop MOF nanosheets, in which Tb/Eu-bop represents 2D MOFs with Tb/Eu as nodes and 5-boronoisophthalic acid as the ligand. Adapted from ref. 133. Copyright 2022, Elsevier Inc.

Another attractive 2D material family is rare earth-containing 2D metal–organic frameworks (MOFs). Compared with traditional 3D MOFs, the 2D MOFs exhibit a larger specific surface area, faster electron transfer and abundant exposed active sites that are accessible, which enable them with improved properties such as electrochemiluminescence (ECL), photocatalysis and sensitivity.^127,128^ With ytterbium as nodes and 4,4′,4′′,4′′′–(21*H*,23*H*-porphine-5,10,15,20-tetrayl) tetrakis-benzoic acid (H_2_TCPP) as the ligand in dimethylformamide (DMF) solution, 2D Yb-MOFs were synthesized and exhibited thickness-dependent ECL behavior. In particular, the thinner the 2D Yb-MOFs, the stronger the ECL signals, which was ascribed to the large surface area, better electrochemical conductivity and higher productive ratio of fluorescence quantum yield.^129^ Furthermore, the porphyrin-based 2D Ln-MOFs, especially porphyrin-based 2D Yb-MOF (namely, Yb-TCPP MOF), display outstanding photodynamic activity (Fig. 7a and b).^130^ By coupling the porphyrin-based 2D Yb-TCPP MOF with methylene blue (MB), Jiang *et al.* prepared 2D artificial light-harvesting-system (MB/Yb-TCPP) nanosheets. The MB/Yb-TCPP nanosheets showed efficient photon capture, energy transfer and various active centres, which led to excellent photocatalytic properties (Fig. 7c and d).^131^ Moreover, rare earth cations such as Eu^3+^ and Tb^3+^ are also introduced into the 2D Zn(ii) MOF to form 2D Ln^3+^-encapsulated functional materials with a tuneable emission color.^134^ With 2,2′-thiodiacetic acid (TDA) as a surfactant, a series of rare earth-based layered MOF can be synthesized, and the layered structures can further be exfoliated into ultrathin nanosheets in EtOH under ultra-sonication conditions (Fig. 7e and f).^132^ Through simple mixing of Tb/Eu salts and 5-boronoisophthalic acid (5-bop) in the presence of triethylamine (TEA) at room temperature, Wang *et al.* synthesized three boric acid-functionalized 2D MOF nanosheets, *i.e.*, Tb-bop, Eu-bop and bimetallic Tb/Eu-bop MOF nanosheets, respectively, expanding the 2D MOF family (Fig. 7g and h).^133^ Though several 2D MOFs containing rare earths have been synthesized in recent years, the ligands used are quite scarce, resulting in limited availability of rare earth-containing 2D MOFs, which deserve more attention and exploration.

2D rare earth oxyhalides (REOXs) represent another interesting class of rare earth-containing 2D compounds. The CVD method is widely used for the preparation of the REOXs. Through this method, Chen *et al.* successfully synthesized EuOCl with an ultra-narrow linewidth of 1.2 meV at room temperature while Tian *et al.* synthesized DyOCl with strong A-type antiferromagnetism below the Néel temperature *T*_N_ of 10 K.^135,136^ Zhang *et al.* designed a facile strategy to prepare a series of 2D LnOCl (Ln = La, Pr, Nd, Sm, Eu, Gd, Tb, Dy) nanoflakes using the molten salt method assisted by the substrate. The lateral size of the prepared ultra-thin nanoplate could reach up to 40 μm with a thickness of only 7.5 nm.^137^ As a gate dielectric, the LnOCl nanoflake exhibited competitive device characteristics of high on/off ratios up to 10^7^ and low subthreshold swings down to 77.1 mV dec^−1^.

### 2.4 Assembly of rare earth-based or doped nanosheets into functional structures

**Fig. 8:**
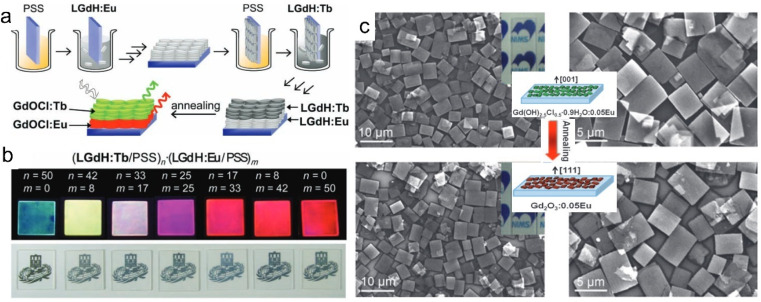
Multilayer rare earth hydroxide films. (a) Scheme of the fabrication process and (b) photographs of multilayer LGdH/PSS films. Adapted from ref. 60. Copyright 2010, John Wiley & Sons, Inc. (c) SEM images and schematic illustration of Gd(OH)_2.5_Cl_0.5_·0.9H_2_O:0.05Eu film and Gd_2_O_3_:0.05Eu film. Reproduced from ref. 138. Copyright 2009, John Wiley & Sons, Inc.

As one kind of nanosheets showing unique properties, rare earth-based or doped nanosheets can be re-assembled into desirable forms for function exploration. Layer-by-layer (LBL) deposition method is a useful method to fabricate the nanosheets on quartz or silicon wafers. As the LREH nanosheets are positively charged, after introducing the negatively charged poly(sodium-*p*-styrenesulfonate) (PSS) ions as counter ions, the LREH nanosheets can be transferred on the wafer and further LBL assembled to form multilayer films, such as (LGdH:Eu/PSS)_*n*_ and (LGdH:Tb/PSS)_*n*_ films, where LGdH:Eu represents Eu^3+^ doped LGdH and LGdH:Tb represents Tb^3+^ doped LGdH (Fig. 8a and b).^60,61^ Through self-assembly at the hexane/water interface, Eu(OH)_2.5_Cl_0.5_·0.9H_2_O and Gd(OH)_2.5_Cl_0.5_·0.9H_2_O:0.05Eu can be transferred upon a glass wafer.^138,139^ After an annealing treatment, the hydroxide film can be quasi-topotactically transformed into Gd_2_O_3_:0.05Eu (Fig. 8c).^138^ The self-assembled film is semi-transparent with a flat surface and strong adhesion, and exhibits high orientation, which is beneficial for enhancement of photoluminescence. This quasi-topotactic transformation and enhanced photoluminescence originating from high orientation were also found in LYH and Y_2_O_3_ films which were fabricated by spin-coating.^140^

**Fig. 9:**
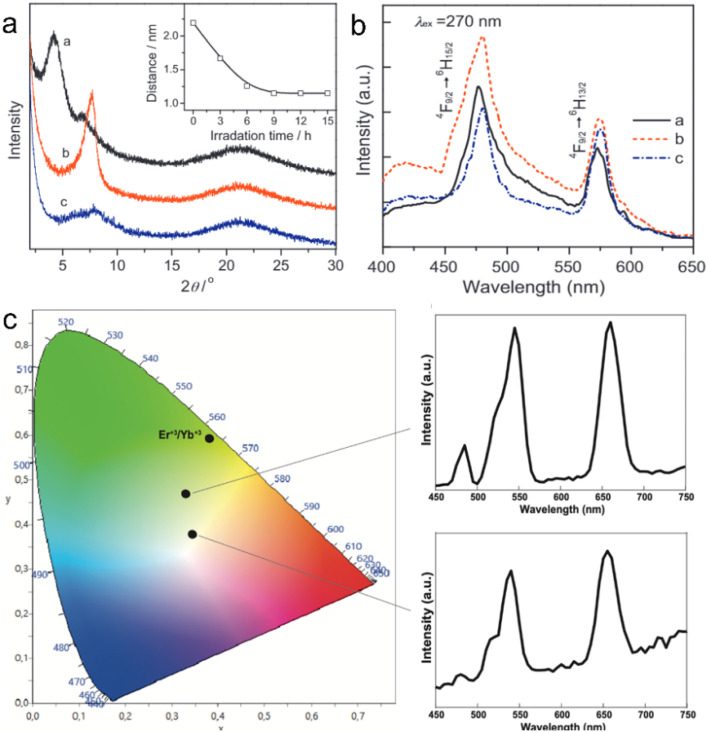
Rare earth perovskite superlattice films through the LBL fabrication method. (a) XRD patterns and (b) photoluminescence emission spectra of (PEI/La_0.90_Dy_0.05_Nb_2_O_7_)_10_ films. Adapted from ref. 123. Copyright 2012, Elsevier Inc. (c) CIE diagram and upconversion emission spectra of (Ln_2_Ti_3_O_10_:Er^3+^,Yb^3+^/Ln_2_Ti_3_O_10_:Tm^3+^,Yb^3+^)_60_ film. Adapted from ref. 125. Copyright 2022, Elsevier Inc.

Through the LBL deposition method, the negatively charged La_0.90_Dy_0.05_Nb_2_O_7_ nanosheets can be self-assembled with polyethylenimine (PEI) as a connector on a quartz, resulting in (PEI/La_0.90_Dy_0.05_Nb_2_O_7_)_*n*_ multilayer films (Fig. 9a). Photoluminescence analysis shows that the as-prepared film displayed a blue-light emission with peaks centred at around 480 and 576 nm (Fig. 9b), and it was found that compared with the direct excitation for the bulk structure, the host excitation is in a dominant state in the nanosheet and film.^123^ Moreover, multilayer upconversion films of alternatingly assembled Er^3+^/Yb^3+^ co-doped Ln_2_Ti_3_O_10_ (Ln_2_Ti_3_O_10_:Er^3+^,Yb^3+^) and Tm^3+^/Yb^3+^ co-doped Ln_2_Ti_3_O_10_ (Ln_2_Ti_3_O_10_:Tm^3+^,Yb^3+^) nanosheet on indium tin oxide (ITO) glass presented a white emission (Fig. 9c).^125^

**Fig. 10:**
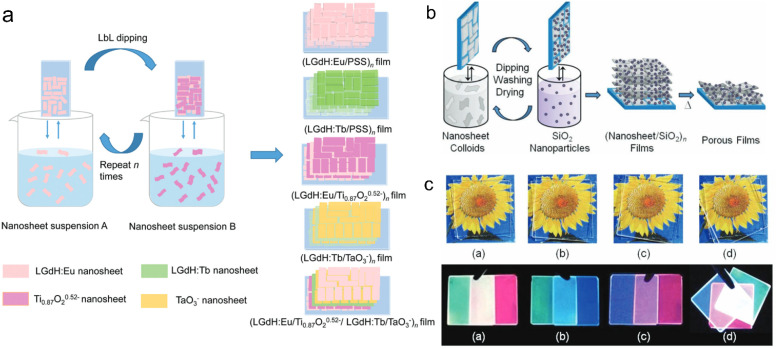
LREH superlattice films through the LBL fabrication method. (a) Scheme of the fabrication process of LREHs/semiconducting oxide superlattice films. Adapted from ref. 61. Copyright 2021, the Royal Society of Chemistry. (b) Scheme of the fabrication process and (c) photographs of (Gd_2_O_3_:Re/SiO_2_) films under daylight and 254 nm UV irradiation, in which Gd_2_O_3_:Re represents Gd_2_O_3_ doped with different rare earth ions. Adapted from ref. 59. Copyright 2012, John Wiley & Sons, Inc.

Assembling the rare earth-containing nanosheets with other 2D nanosheets through the LBL method, superlattice-like nanofilms were fabricated. Ida *et al.* reported a bilayer film composed of Eu(OH)_3−*x*_ nanosheets and Ti_1.81_O_4_ nanosheets with drastically enhanced luminescence.^141^ Bai *et al.* further fabricated a series of superlattice films with LREH nanosheets and semiconductive nanosheets, such as (LGdH:Eu/Ti_0.87_O_2_^0.52−^)_*n*_ films, (LGdH:Tb/TaO_3_^−^)_*n*_ films and (LGdH:Eu/Ti_0.87_O_2_^0.52−^/LGdH:Tb/TaO_3_^−^)_*n*_ films and systematically studied the energy transfer process within the superlattice films, in which LGdH:Eu and LGdH:Tb represent Eu^3+^ or Tb^3+^ doped LGdH, respectively (Fig. 10a).^61^ When LREH nanosheets were assembled with SiO_2_ nanoparticles, LREHs/SiO_2_ porous multilayer films were prepared with high transparency (Fig. 10b and c).^59,142^ The emission light color could be tuned by adjusting the composition of the different color light sources. Therefore, the LBL method is a useful way to fabricate multilayer films with controllable compositions and structures by using ultra-thin nanosheets. In addition, through a block-by-block epitaxial growth of the multistep molten salt method, SmOCl-NdOCl-EuOCl multi-heterostructure and SmOCl-NdOCl-SmOCl-NdOCl superlattice films were fabricated, that is, growth of the second and subsequent layers by precisely sprinkling the powders beside the existing crystals under the same conditions.^137^

**Fig. 11:**
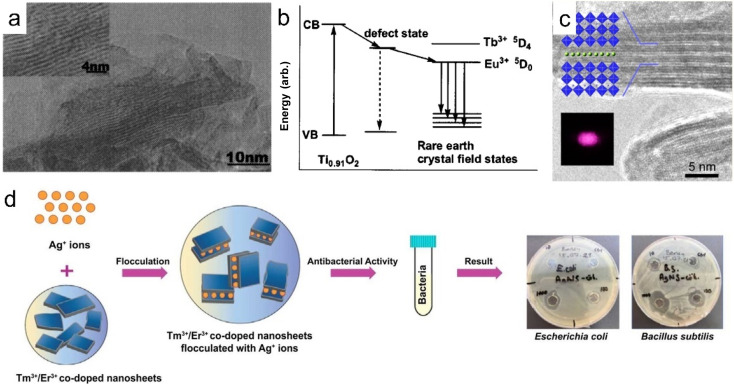
Rare earth-doped perovskite lamellar flocculation. (a) TEM image and (b) possible model of the energy diagram of Eu^3+^ flocculated Ti_0.92_O_2_ nanosheet (ex-Ti_0.92_O_2_/Eu) composite. Adapted from ref. 143. Copyright 2004, American Institute of Physics. (c) TEM image of (Ho_0.096_Yb_0.23_Y_0.164_)Ca_1.76_Nb_3_O_10_ composite. Adapted from ref. 144. Copyright 2014, American Chemical Society. (d) Fabrication process and growth inhibition of Ag^+^ intercalated Tm^3+^/Er^3+^ co-doped Ln_2_Ti_3_O_10_ composite. Adapted from ref. 70. Copyright 2022, Elsevier Inc.

A clean substrate (*e.g.*, quartz glass or silicon wafer) is generally needed for the above-mentioned LBL assembly method, and the rare earth-containing multilayer films assembled by this method are widely used in the luminescence field. As rare earth-doped perovskite nanosheets are negatively charged, they can also be flocculated by positively charged cations to form lamellar structures. This flocculation method endows rare earth-based or doped nanosheets with more properties. Xin *et al.* flocculated negatively charged Ti_0.91_O_2_ nanosheets with Eu^3+^ or Tb^3+^ ions to form ex-Ti_0.92_O_2_/Eu and ex-Ti_0.92_O_2_/Tb composites, respectively, and found that the flocculation was in a lamellar structure with a gallery distance of 1.06 nm (Fig. 11a).^143^ The photoluminescence analysis indicates that nonradiative energy transfer from the Ti_0.91_O_2_ host to Eu^3+^ can take place in this system (Fig. 11b).^143^ Similarly, red- and green-light-emitting Eu^3+^ or Tb^3+^ ion-flocculated TiTaO_5_ lamellar aggregates were prepared.^145^ Through flocculating Ca_2_Nb_3_O_10_ nanosheets with Ho^3+^, Yb^3+^ and Y^3+^ ions, Ozawa *et al.* prepared a new upconversion lamellar material (Ho_0.096_Yb_0.23_Y_0.164_)Ca_1.76_Nb_3_O_10_, in which Ca_2_Nb_3_O_10_ nanosheets were restacked in a parallel manner with three layers (Fig. 11c).^144^ While using Ag^+^ as the counter cations, Günay *et al.* fabricated an Ag-intercalated Tm^3+^/Er^3+^ co-doped Ln_2_Ti_3_O_10_ composite, and found that the flocculation exhibited efficient antibacterial and antibiofilm activity but also low cytotoxicity (Fig. 11d).^70^ Compared with the LBL fabrication method, this flocculation method shortens the time largely and can enhance or expand the properties of nanosheets by the introduction of counter ions, but it lost the superlattice-like structures.

**Fig. 12:**
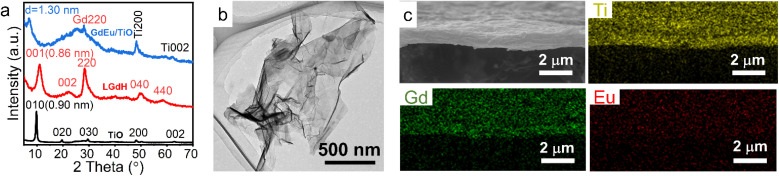
(a) XRD patterns, (b) TEM image and (c) SEM and elemental mapping images of the GdEu/TiO membrane, in which GdEu and TiO represent Eu^3+^ doped LGdH nanosheets and Ti_0.87_O_2_^0.52−^ nanosheets, respectively. Adapted from ref. 146. Copyright 2022, John Wiley & Sons, Inc.

Based on electrostatic absorption, Bai *et al.* constructed a self-standing superlattice membrane through the direct assembly of LREH nanosheets and 2D semiconductive oxide counterparts in a face-to-face manner (Fig. 12).^34^ The emission light color of the (GdEu/TiO)/(GdTb/TaO) membrane can be controlled by adjusting the excitation wavelength numbers, where GdEu and GdTb represent Eu^3+^ or Tb^3+^ doped LGdH nanosheets, respectively, TiO and TaO represent Ti_0.87_O_2_^0.52−^ and TaO_3_^−^ nanosheets, respectively. In addition, the self-standing membrane exhibited stable cathode luminescence under continuous electron bombardment. When the LREH nanosheets were assembled with Ti_3_C_2_ nanosheets, the LREH/MXene hybrid presented excellent photothermic and MRI properties.^146^ This re-assembly method of nanosheets will inspire the structural design development of rare earth-based or doped 2D materials with multifunction.

All the above assembly methods of ultrathin rare earth-based or doped nanosheets, especially for the superlattices, inspired new strategies to integrate different nanosheets and promote the design of rare earth nanomaterials towards novel structures and interesting functionalities. Further efforts in the assembly of more functional nanosheets with different compositions/structures are needed to enhance and expand the applications of rare earth nanomaterials.

## 3. Biomedical applications based on 2D rare earth nanomaterials and composites

The properties of rare earth-containing nanomaterials are largely affected by their structures and doping elements. Tables 1–3 summarize the morphology, rare earth-containing elements and functions of 2D rare earth nanomaterials. It is obvious that the layered structure with interlayer ion exchangeability is beneficial in drug release, especially for LREHs. On the other side, due to the half-filled 4f electrons, the existence of Gd^3+^ endows the nanomaterials with magnetic properties, resulting in their application as MRI agents. Moreover, for ultrathin nanosheets, the abundant active side ensures that it can be modified with other functional nanosheets/groups, thus expanding their application scenarios. This section summarizes the composition, general properties and applications of 2D rare earth nanomaterials.

**Table 1:** Summary of 2D rare earth nanomaterials for drug delivery applications

Materials	Morphology	Drugs	Ref.
Y/Tb/Gd-LREH	Layered nanocone	ASA	44
LTbH-Cl	Layered plate	Dic, ibu, nap	46
LGdH-Cl	Layered plate	Dic, ibu, nap	57
LTbH-NO_3_	Layered plate	ASA	58
LEuH-NO_3_	Layered plate	Nap	106
LGdH-NO_3_	Layered plate	Nalidixic acid, aspartic acid, glutamic acid, fatty acid	107
LGdH-Cl	Layered plate	Anti-miRNA oligonucleotides	108
PPF-Gd MOF	Layered plate	Dox	147
Gd&Yb-LDH	Nanosheet	Chemotherapeutic drug (SN38)	148

**Table 2:** Summary of 2D rare earth nanomaterials for bioimaging applications

Materials	Rare earth elements	Bioimaging applications	Ref.
LGdH	Gd	MRI	57
LGdH/Ti_3_C_2_	Gd	MRI	146
GdDy-LDH	Gd	MRI	149
GdCu-LDH	Gd	MRI	150
LDH-Gd(dtpa)	Gd	MRI	151 and 152
AFGd-LDH	Gd	MRI	153
LGdH:Ce,Tb	Gd, Ce, Tb	MRI, CT	52
UCSP-FeMn-LDH	Gd, Yb, Er	MRI, CT	154
PPF-Gd	Gd	MRI, fluorescence imaging	147
FITC/FA-DOX/Gd-LDH	Gd	MRI, fluorescence imaging	155
Ce6&AuNCs/Gd-LDH	Gd	MRI, fluorescence imaging	156
Gd/MgGa-LDH	Gd	MRI, CT imaging	157
SN38&ICG/Gd&Yb-LDH	Gd, Y	MRI, CT imaging, NIRF imaging	148
ICG/CAC-LDH	Gd	MRI, photoacoustic imaging	158

**Table 3:** Summary of 2D rare earth nanomaterials for tumor therapy applications

Materials	Tumor therapy applications	Ref.
Phy@PLGdH	Radiation therapy	62
PPF-Gd/DOX	Chemotherapy	147
LGdH/Ti_3_C_2_	PTT	146
SN38&ICG/Gd&Yb-LDH	PTT	148
BOSC	PCT	159 and 160
NaYF_4_:Yb,Tm@NaGdF_4_	PDT	161
ICG/CAC-LDH	PTT, CDT	158
DOX&ICG/MLDH	Chemotherapy, PTT, PDT	162
UCSP-FeMn-LDH	PTT, PDT, CDT	154

### 3.1 Drug delivery

For layered structured nanomaterials, the abundant interlayer space and rich interlayer ion exchangeability greatly promote their drug load and release properties, which provide more ideas for the design of drug delivery carriers.

**Fig. 13:**
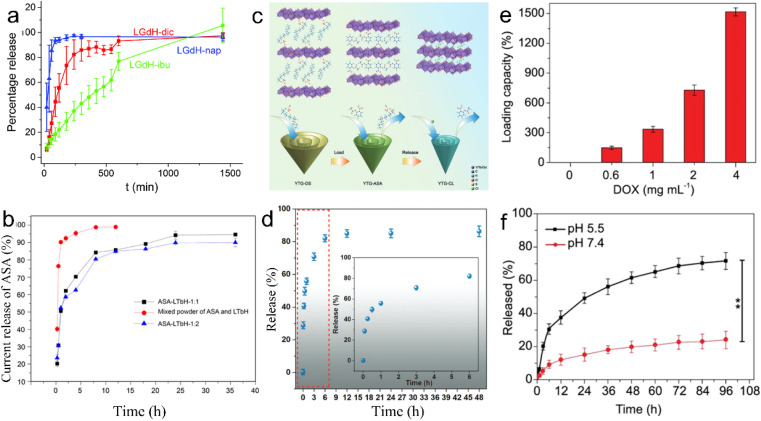
Release kinetics of (a) diclofenac, ibuprofen, and naproxen from LGdH. Adapted from ref. 57 Copyright 2018, the Royal Society of Chemistry. (b) Release kinetics of aspirin from LTbH, in which ASA-LTbH-1 : 1 and ASA-LTbH-1 : 2 represent ASA intercalated LTbH at a molar ratio of ASA to NaOH of 1 : 1 or 1 : 2, respectively. Reproduced from ref. 58. Copyright 2017, John Wiley & Sons, Inc. (c) Scheme of drug loading and release process and (d) release kinetics of ASA from Y/Tb/Gd ternary layered rare earth hydroxide nanocones, in which YTG-DS, YTG-ASA and YTG-CL represent DS^−^, ASA and Cl^−^ intercalated Y/Tb/Gd ternary layered rare earth hydroxide nanocones, respectively. Reproduced from ref. 44. Copyright 2023, John Wiley & Sons, Inc. (e) Drug loading and (f) release capacity of the Gd-based porphyrin paddlewheel framework (PPF-Gd). Adapted from ref. 147. Copyright 2020, Oxford University Press.

Through anion exchange or direct synthesis, various drugs, such as diclofenac (dic), ibuprofen (ibu), naproxen (nap), aspartic acid, glutamic acid, palmitic acid, anti-miRNA oligonucleotides and aspirin (ASA), can be intercalated into the gallery of the LREHs.^46,57,58,106–108^ The non-steroidal anti-inflammatory drugs (diclofenac, ibuprofen, and naproxen) can be inserted into LGdH, and the LGdH-drug intercalates are stable in neutral pH and degrade rapidly under acidic conditions.^57^ Different drugs exhibit diverse release times. For example, naproxen-intercalated LREH releases the drug loadings very rapidly (within 1.5 h), and the diclofenac-intercalated one shows sustained release time over 4 h, while the ibuprofen-intercalated counterpart shows a continuous release time of 24 h and the ASA-loaded LREH extends the release time up to 36 h, as shown in Fig. 13a and b.^57,58^ Furthermore, the LREHs-drug composites show good targeted drug delivery properties, high drug loading and low cytotoxicity, enabling the LREHs with potential applications in drug delivery. Gu *et al.* prepared a 5-aminolevulinic acid (5-ALA) intercalating LREH-coated MgFe_2_O_4_ particles (5-ALA-MgFe_2_O_4_@LREH). With the help of magnetic MgFe_2_O_4_, the 5-ALA-MgFe_2_O_4_@LREH carries drugs efficiently to targeted locations under an external magnetic field.^45^ Recently, Li *et al.* proposed Y/Tb/Gd ternary LREH nanocones with drug delivery and simultaneous MRI properties, as shown in Fig. 13c.^44^ Through anion exchange, ASA can be loaded into the gallery with a delayed release behavior to 48 h (Fig. 13d), which is favorable for sustained therapy. As the intercalation of ASA can enhance the green light emission at the peak of 543 nm, the nanocones can also be utilized as a fluorescence probe to monitor the loading and release process of ASA. Furthermore, the ASA intercalation improves the magnetic properties of the nanocones, which enables an additional application as an MRI contrast agent simultaneously.

In addition to inorganic 2D nanomaterials, rare earth-porphyrin MOF nanosheets can also serve as drug delivery materials. Through the surfactant-assisted solvothermal method, a series of lanthanide-porphyrin MOF nanosheets (denoted as PPF-Ln) with ultrahigh drug loading capacity were prepared.^147^ The results show that the loading capacity of PPF-Gd nanosheets can reach 1515% when the doxorubicin (DOX) concentration is 4 mg mL^−1^ (Fig. 13e). Also, PPF-Gd nanosheets exhibit pH-responsive biodegradation and persistent drug release performance. As shown in Fig. 13f, in the phosphate buffered saline (PBS) solution (pH = 5.5), about 49% DOX was released over 24 h, while only 15% DOX was released at pH 7.4. The final release percentage reached 72% and 24% at pH 5.5 and 7.4 after 96 h, respectively. Moreover, the *in vivo* results confirm the evident suppression of the tumor growth by the PPF-Gd/DOX system with negligible side effects. Owing to the unique layered structure, these 2D layered rare earth nanomaterials hold controllable drug load and release properties, the release time varies from within 1.5 h to several days depending on the type of drug and environment pH, which enables the 2D rare earth nanomaterials to have promising drug delivery applications in different *in vivo* environments.

Apart from those described above, 2D rare earth nanomaterials can also load and release other therapeutic drugs. The drug delivery applications are summarized in Table 1. Despite the promising commercial prospects, 2D rare earth nanomaterials as drug delivery agents still have some deficiencies. For instance, LREHs as typical hydroxides are stable in neutral and alkaline systems, but are easily decomposed in acidic systems. Thus they are scarcely used in acidic environments such as in the stomach, which greatly limits their application prospects. Moreover, since LREHs and perovskite host layers are positively and negatively charged, respectively, they exhibit good drug load properties for the negatively and positively charged drugs. However, for those drugs with the same electrical charge or neutral property, their loading capacity needs to be improved.

### 3.2 Bioimaging

Magnetic resonance imaging, computed tomography (CT), B-scan ultrasonography and X-ray radiography are the most common medical imaging diagnostic technologies. The diverse 4f electron shell endows rare earth-containing nanomaterials with unique magnetic properties. For example, Sc^3+^, Y^3+^, La^3+^ and Lu^3+^ are magnetically inert and are mainly employed as host cations for doping with other ions due to their empty or completely filled 4f electrons.^163^ On the other hand, most rare earth ions are paramagnetic at room temperature due to the unpaired 4f electrons, such as Ce^3+^, Gd^3+^, Tb^3+^ and Eu^3+^, in which Gd^3+^ possesses the largest magnetic moment of all rare earth ions due to the highly symmetric ground state and seven unpaired 4f electrons.^163–166^ Thus, Gd^3+^-containing nanomaterials, with abundant unpaired electrons of the high-spin states of Gd^3+^, exhibit significant magnetic properties and are promising MRI agents for the detection of pathological tissues.^41,42,108,167^

**Fig. 14:**
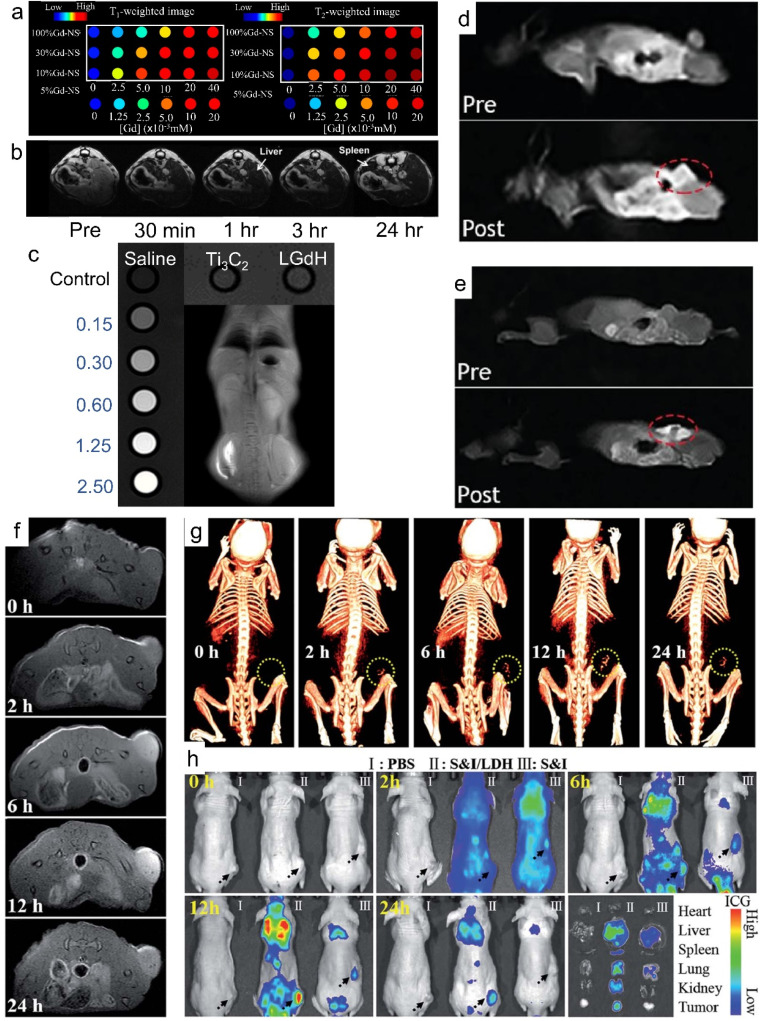
MRI properties of Gd^3+^-containing 2D nanomaterials. (a) *T*_1_-weighted and *T*_2_-weighted phantom images of Gd/Y hydroxide nanosheets. Reproduced from ref. 43. Copyright 2020, Multidisciplinary Digital Publishing Institute. (b) *In vivo T*_2_-weighted MRI of mouse body with intravenous injection of LGdH-FS-PEGP, in which LGdH-FS-PEGP represents poly(ethylene glycol)-phospholipid-modified LGdH. Adapted from ref. 42. Copyright 2009, John Wiley & Sons, Inc. (c) *In vitro* and *in vivo T*_1_-weighted MRI effect of LGdH/Ti_3_C_2_. Adapted from ref. 146. Copyright 2023, the Royal Society of Chemistry. *In vivo T*_1_-weighted MR coronal images of a nude mouse bearing A375 tumor before and after (d) intravenous injection and (e) subcutaneous injection of PPF-Gd nanosheets, where PPF-Gd represents Gd-porphyrin MOF nanosheets. Adapted from ref. 147. Copyright 2020, Oxford University Press. (f) *In vivo* MR and (g) CT imaging of nude mice bearing HeLa tumors at different time points after intravenous injection of SN38&ICG/Gd&Yb-LDH. (h) *In vivo* fluorescence imaging and drug bio-distribution for nude mice bearing HeLa tumors at different time points after intravenous injection of PBS, SN38&ICG/Gd&Yb-LDH and SN38&ICG, respectively, together with *ex vivo* imaging of ICG in the tumour and five different organs collected from mice sacrificed at 24 h, in which SN38&ICG/Gd&Yb-LDH represents a chemotherapeutic drug (SN38) and indocyanine green (ICG) co-modified Gd^3+^ and Yb^3+^ co-doped LDH. Adapted from ref. 148. Copyright 2018, the Royal Society of Chemistry.

As shown in Fig. 14a, Gd/Y hydroxide nanosheets show excellent MRI performance with high longitudinal and transverse relativities of *r*_1_ (103 mM^−1^) and *r*_2_ (372 mM^−1^) (Fig. 14a).^43^ When modified with phospholipids, LGdH nanosheets can work as an efficient MRI contrast agent and show a significant signal loss in the *T*_2_-weighted MRI of the abdomen (Fig. 14b).^42^ The intercalation of some drugs (*e.g.*, diclofenac, ibuprofen, and naproxen) can further enhance the MRI performance of LREHs.^57^ While MRI contrast agents generally show low contrast efficacy for MRI systems with ultrahigh magnetic field higher than 7.0 T, Ce and Tb co-doped LGdH (denoted as LGdH:Ce,Tb) shows excellent negative (*T*_2_) contrast agent efficacy for 7.0 T MRI with a high *r*_2_/*r*_1_ ratio of 48.80, whereas a lower *r*_2_/*r*_1_ ratio of 30.60 for 3.0 T MRI, indicating a high-performance *T*_2_-weighted contrast agent in ultrahigh-field MRI.^52^ Furthermore, the LGdH:Ce,Tb can also work as a contrast agent in CT and fluorescence bioimaging. In recent years, our team proposed several studies on LREH-based nanomaterials with MRI properties. Though the half-filled 4f electron configuration of Gd^3+^ endows the Gd^3+^-containing nanomaterials with MRI properties, it's negligible for the pure layered Y/Gd hydroxide. Though the intercalation of ASA can enhance the *T*_1_-weighted *in vitro* MRI performance, the *in vivo* MRI effects are still not obvious enough.^44^ Through the construction of a superlattice of LGdH nanosheets and Ti_3_C_2_ nanosheets, Bai *et al.* proposed an LGdH/Ti_3_C_2_ hybrid, which exhibited obvious enhanced *in vitro* and *in vivo T*_1_-weighted MRI effect, as shown in Fig. 14c.^146^

Through introducing guests or anion exchange, Gd^3+^ and Gd^3+^-based contrast agents are doped into LDH to improve their MR relaxation.^168^ Gd^3+^ and Dy^3+^ co-doped LDH (GdDy-LDH) showed high contrast effect in MRI with *r*_1_ = 3.49 mM^−1^ s^−1^ and *r*_2_ = 18.17 mM^−1^ s^−1^.^149^ When Gd^3+^ and Cu^2+^ are co-doped in LDH, the GdCu-LDH showed significantly higher longitudinal relativity compared with those solely doped with Gd^3+^ or Cu^2+^ (Gd-LDH or Cu-LDH) due to the synergistic *T*_1_ shortening between adjacent Gd^3+^ and Cu^2+^ in the LDH host layers.^150^ Through ion exchange, Xu *et al.* prepared Gd-dtpa (dtpa = diethylene triamine pentaacetate) intercalated Mg_2_Al-Cl-LDH (LDH-Gd(dtpa)).^151,152^ Compared to Gd(dtpa) free LDH, the LDH-Gd(dtpa) displayed 4 times increase in longitudinal proton relaxation and a 12 times increase in transverse proton relaxation.^151^ Guan *et al.* developed a supramolecular nanomaterial (FITC/FA-DOX/Gd-LDH) *via* co-intercalation of folic acid (FA) and DOX into the Gd-LDH, followed by adsorption of fluorescein isothiocyanate (FITC), FITC/FA-DOX/Gd-LDH exhibited excellent *T*_1_-weighted MRI and fluorescence dual-mode imaging activity.^155^ Through adding gold nanoclusters (AuNCs) and Chlorin e6 (Ce6) to the Gd-LDH, Mei *et al.* synthesized Ce6&AuNCs/Gd-LDH. Both *in vitro* and *in vivo* results confirmed that the Ce6&AuNCs/Gd-LDH showed MRI and fluorescence dual-mode imaging activity.^156^

Other than inorganic 2D materials, rare earth-based 2D MOFs show promising applications in the bioimaging field.^169,170^ The PPF-Gd nanosheets exhibit an obviously enhanced *T*_1_-weight MR signal with a transverse relativity of 10.04 mM^−1^ s^−1^.^147^ As shown in Fig. 14d and e, for both intravenous injection and subcutaneous injection, the *in vivo T*_1_-weighted MRI signal is detected and significantly higher than that before injection. Moreover, the PPF-Gd nanosheets show obvious fluorescence imaging properties following subcutaneous injection after 72 h.

As summarized in Table 2, 2D rare earth nanomaterials can also work as CT imaging and NIR imaging agents in addition to MRI. Through reverse micelle preparation of LDH and Gd(OH)_3_, Jung *et al.* synthesized Gd/MgGa-LDH hybrids, which exhibited CT and MR dual-modal imaging performance, due to the high X-ray attenuation coefficient and paramagnetic properties of Gd^3+^ in Gd(OH)_3_.^157^ Mei *et al.* prepared Gd^3+^ and Yb^3+^ co-doped LDH (Gd&Yb-LDH) monolayer nanosheets. The experiments on the *in vivo* metabolic pathway indicated that *in vivo* tri-mode (MR, CT and near-infrared fluorescence (NIRF)) imaging was achieved (Fig. 14f–h), enabling a non-invasive visualization of cancer cell distribution with deep spatial resolution and high sensitivity.^148^ The Gd&Yb-LDH monolayers exhibited ultrahigh loading content of a chemotherapeutic drug (SN38) up to 925%. Furthermore, SN38&ICG/Gd&Yb-LDH was prepared by further modifying Gd&Yb-LDH with SN38 and ICG. As shown in Fig. 14f and g, after intravenous injection of SN38&ICG/Gd&Yb-LDH, the tumor site of nude mouse displayed a gradually enhanced MRI and CT imaging signal due to the enhanced permeability and retention (EPR) effects and preferential accumulation, respectively. As shown in Fig. 14h, after the injection of SN38&ICG and SN38&ICG/Gd&Yb-LDH for 2 h, significant NIRF signals were observed throughout the whole body. For the injection of SN38&ICG/Gd&Yb-LDH, the signal remained after 24 h while it was rather weak for SN38&ICG after 6 h.

Thus, both the inorganic and organic Gd^3+^-containing 2D nanomaterials exhibit high potential in bioimaging applications. Although extensive work has been conducted on the magnetic property modulation and MRI applications of 2D rare earth nanomaterials, it mainly focuses on Gd^3+^-containing materials. Other RE-based nanomaterials have been rarely explored, which needs more study.

### 3.3 Tumor therapy

On account of biocompatibility and low toxicity, the applications of 2D rare earth nanomaterials in other biochemical fields beyond drug delivery and MRI contrast agents have also been explored recently, especially tumor therapy.

**Fig. 15:**
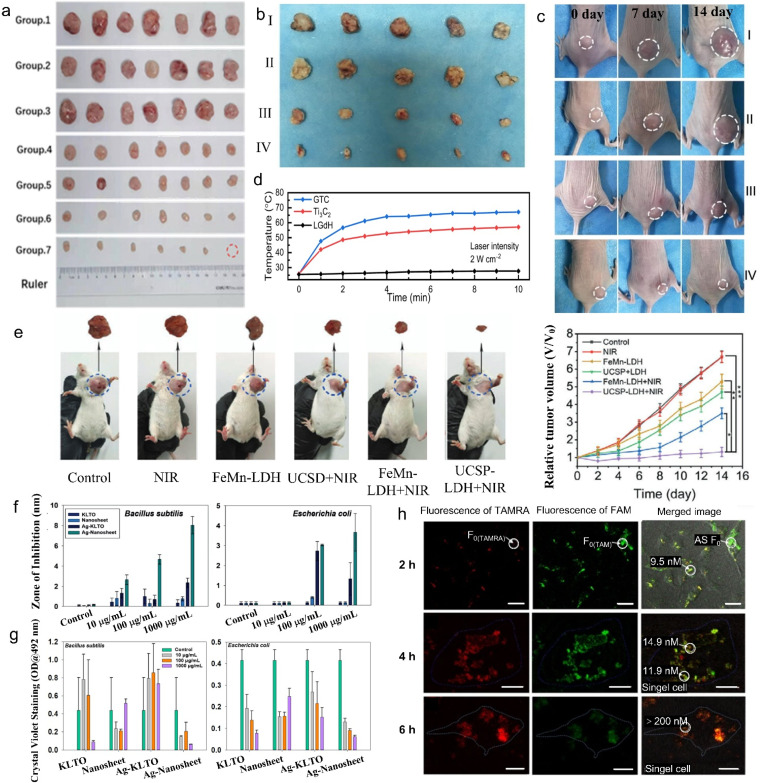
Tumor therapy, antibacterial activity and living cell sensing properties of 2D rare earth nanomaterials. (a) Images of CT-26 tumor tissues collected on day 15 after being treated with saline, PLGdH, Phy@PLGdH, saline + RT, Gd-NCPs + RT, PLGdH + RT, Phy@PLGdH + RT (group 1–7), in which PLGdH represents PEG-modified LGdH, Phy@PLGdH represents physcion modified PLGdH, Gd-NCPs represents spherical Gd-based nanoscale coordinate polymers and RT represents radiation therapy. Adapted from ref. 62. Copyright 2022, John Wiley & Sons, Inc. Photographs of (b) tumors from different groups after 14 days treatment and (c) representative tumors in mice from different groups, in which group I to IV represent phosphate-buffered saline (PBS), gadolinium-based porphyrin paddlewheel framework (PPF-Gd), doxorubicin (DOX) and DOX modified PPF-Gd (PPF-Gd/DOX) treatment, respectively. Reproduced from ref. 147. Copyright 2020, Oxford University Press. (d) Heating curves of the LGdH/Ti_3_C_2_ hybrid under NIR laser irradiation. Adapted from ref. 146. Copyright 2023, the Royal Society of Chemistry. (e) Representative digital photographs of excised tumor invidious treating groups of tumor-bearing mice after 14 days' treatment. (g) The relative tumor volume of tumor-bearing mice treated under different conditions. Adapted from ref. 154. Copyright 2020, John Wiley & Sons, Inc. (f) Antibacterial properties and (g) antibiofilm properties of Tm^3+^/Er^3+^ co-doped K_2_La_2_Ti_3_O_10_ nanomaterials before and after Ag^+^ intercalation. Adapted from ref. 70. Copyright 2022, Elsevier Inc. (h) Confocal images of intracellular visualization for adenosines after treatment with the dye (tetramethylrhodamine (TAMRA) or fluorescein (FAM))-labelled MOF–La complex after an incubation time of 2 to 6 h and quantification of adenosines by two-color fluorescence. Adapted from ref. 132. Copyright 2017, Springer Nature.

For instance, poly(ethylene glycol) (PEG) modified LGdH (PLGdH) presents superior X-ray deposition and tumor penetrability. Moreover, after further encapsulation of physcion (Phy), the Phy-modified PLGdH (Phy@PLGdH) can further amplify PLGdH-sensitized room temperature mediated oxidative stress and DNA damage, and synergistically induce the potent immunogenic death, resulting in highly active tumor inhibition in radiation therapy (RT) (Fig. 15a).^62^ In addition, Gd-based porphyrin paddlewheel framework (PPF-Gd) MOF showed significantly high *in vitro* and *in vivo* tumor inhibition performance, as illustrated in Fig. 15b and c. After subcutaneous injection, the doxorubicin (DOX) modified PPF-Gd (PPF-Gd/DOX) exhibited a greatly enhanced inhibition efficacy to the tumor growth.^147^ Meanwhile, the weight of mice was slightly increased during the treatment in the PPF-Gd/DOX group, making it an excellent chemotherapy agent candidate in cancer therapy with enhanced therapeutic efficiency and low toxic side effects.

Though RT and thermotherapy are widely used in tumor therapy, they are harmful to biological tissues. Compared with these harmful treatments, PTT treatment has gained increasing attention due to its deep penetration and low tissue damage. For photothermal therapy, the LGdH/Ti_3_C_2_ hybrid exhibited an enhanced photothermal effect under 2 W cm^−2^ irradiation for 10 min, in which the temperature surpassed 60.4 °C (Fig. 15d).^146^ The above-mentioned SN38&ICG/Gd&Yb-LDH demonstrated excellent PTT properties. After the injection of 3 min, the temperature of the nude mice tumor site increased to 51 °C, and the dynamic tumor growth experiment also confirmed the benefit of SN38&ICG/Gd&Yb-LDH to *in vivo* photothermal therapy.^148^

Other than traditional phototherapy and chemotherapy, 2D rare earth nanomaterials were also used in photocatalytic therapy (PCT), photodynamic therapy (PDT) and chemodynamic therapy (CDT).^171,172^ Zhang *et al.* synthesized Ce and S co-doped Bi_2_O_3_ (BOSC) nanosheets with enhanced PCT under near-infrared light irradiation. The introduction of Ce promotes the generation of reactive oxygen species (ROS), which enhances light absorption, introduces oxygen vacancies, reduces bandgap, and facilitates charge separation. The Ce doping also modifies the band position and Fermi level of BOSC, resulting in increased band bending at the solid–liquid interface, enabling a cascade reaction of ROS.^159,160^ On the other hand, Wang *et al.* prepared AFGd-LDH through surface modification of Gd-LDH with atorvastatin (ATO) and ferritin heavy subunit (FTH). Both *in vitro* and *in vivo* experiments verified that the AFGd-LDH shows outstanding MRI performance and alleviates brain reperfusion injury issue due to the excellent ROS scavenging efficiency.^153^

Moreover, PDT has become a promising cancer treatment approach with superior advantages.^161^ Liu *et al.* synthesized a tumor target agent through modifying NaYF_4_:Yb,Tm@NaGdF_4_, folic acid (FA), carboxymethyl triphenylphosphine (TPP) on graphene oxide quantum dots, in which the upconversion nanoparticles serve as a transducer of excitation light.^173^ Both *in vitro* and *in vivo* experiments show that the synthesized nanomaterials exhibited significantly enhanced subcellular targeting and PDT efficacy for cancer therapy.

To further improve the treatment efficiency of tumor cells, researchers have designed 2D rare earth nanomaterials with multiple treatment pathways. Wang *et al.* designed Ce-doped CuAl-LDH (denoted as CAC-LDH) nanosheets and further loaded them with indocyanine green (ICG) to synthesize ICG/CAC-LDH.^158^ The synthesized ICG/CAC-LDH could induce intracellular glutathione (GSH) depletion and reduce Cu^2+^ and Ce^4+^ to Cu^+^ and Ce^3+^, respectively, and further decompose H_2_O_2_ to cytotoxic ·OH through the Fenton reaction. The obviously enhanced absorption at 808 nm and efficient NIR light-to-heat conversion enable the ICG/CAC-LDH with photothermal efficiency and further increase the Fenton reaction rate. Thus, the ICG/CAC-LDH showed remarkable dual-model PTT/CDT efficacy against HepG2 cancer cells. Furthermore, ICG/CAC-LDH can also work as an MRI and photoacoustic imaging (PAI) agent. Moreover, triple-model combined therapy, so-called chemo/PTT/PDT, can be achieved by co-modification of DOX, ICG and Gd^3+^ on MgAl-LDH (denoted as DOX&ICG/MLDH).^162^ Jia *et al.* synthesized UCSP-FeMn-LDH through modifying FeMn-LDH with mesoporous silica and Ce6 co-coated NaGdF_4_:Yb,Er@NaGdF_4_:Yb (UCSP). The UCSP-FeMn-LDH showed excellent oxygen-elevated PDT, enhanced PTT and CDT synergistic therapy (Fig. 15e) and real-time monitoring of therapeutic effect *via* feasible CT imaging and *T*_1_/*T*_2_-weighted MRI.^154^

In addition to tumor therapy, 2D rare earth nanomaterials can also work in other biomedical applications. The Ag^+^-modified Tm^3+^/Er^3+^ co-doped K_2_La_2_Ti_3_O_10_ layered compound demonstrates efficient antibacterial and antibiofilm activity (Fig. 15f and g).^70,122^ Moreover, 2D MOFs with rare earth ions as nodes (MOF-Ln), such as MOF-La, also show some interesting properties in biomedical applications. Wang *et al.* found that the fluorescence of negatively charged fluorescein (FAM)-labelled single-stranded DNA (ssDNA) was partially quenched by MOF-Ln nanosheets, while that of positively charged tetramethyl rhodamine (TAMRA)-labelled ssDNA showed the same quenching and recovery properties on MOF-Ln nanosheets. Thus the MOF-Ln can be used as a two-color sensing platform for intracellular detection in living cells (Fig. 15h).^132^

The summary of 2D rare earth nanomaterials for tumor therapy applications is listed in Table 3. The *in vitro* and *in vivo* experiments verified that 2D rare earth-containing nanomaterials have low cytotoxicity and great biocompatibility.^43,44,62,153^ For example, Phy@LGdH showed no obvious cytotoxicity in CT26 tumor cells and the BOSC showed no significant inhibition of 4T1 mouse breast cancer cells after 48 h of incubation, while the LTbH showed no significant cytotoxicity on Caco-2, HEK 293 cell line, HeLa, fibroblast and rhabdomyosarcoma cancer cell lines.^46,62,148,159^ However, only limited experiments on *in vivo* metabolic pathways have been carried out. More attention in this regard is needed in future clinical applications. Therefore, with the combined advantages of high biocompatibility, low toxicity and the unique biochemical properties of 2D rare earth nanomaterials and composites, further investigations of more 2D rare earth nanomaterials in biomedicine fields, especially in *in vivo* applications, are highly desired.

## 4. Conclusions

In summary, this work provides an overview of the recent progress in the design, preparation and biomedical applications of 2D nanomaterials based on rare earth elements. In particular, focus on the exfoliation of LREHs and rare earth-doped layered perovskites is presented. Through exfoliation, bulk layered LREHs or rare earth-doped perovskites can be dimensionally reduced into ultrathin nanosheets, which promotes further assembly and multifunctional applications. Artificial superlattice assembly is an effective method to enhance the properties of rare earth-based or rare earth-containing nanosheets. Moreover, the application fields can be expanded with integration with other functional counter nanosheets. Progress in diverse biomedical applications (*e.g.*, drug delivery, MRI, cancer therapy, *etc.*) implies that the 2D rare earth nanomaterials have great potential in health-related field applications. Although significant breakthroughs have been made in these fields, some limitations and challenges still exist. Herein, based on the current progress, several prospects and opportunities for the development of 2D nanomaterials based on rare earth are proposed.

(i) It is desirable to expand the family of rare earth-based or doped nanosheets. Diverse-high-quality nanosheets based on rare earth elements with tuneable composition are essential for further research, although a variety of rare earth nanosheets such as LREHs and rare earth-doped perovskite nanosheets have been proposed. Compared with the large class of similar LDHs and perovskites, the class of rare earth-based or doped nanosheets is still limited. Thus, the design and preparation of new 2D nanomaterials based on rare earth elements are of great importance.

(ii) Further studies on the exfoliation process and in-depth understanding of the mechanism are urgent. High quality (*i.e.*, high crystallinity, tuneable lateral size and ultrathin thickness) of rare earth-based or doped nanosheets is vital to promote the assembly and further applications. Although great breakthroughs have been made in the preparation of rare earth-based or doped nanosheets recently, the yield is very low. Furthermore, the nanosheets are unstable in the exfoliation solution, that is, the nanosheets are easily restacked to form flocculations and/or degrade in the solution upon long time storage. Thus, more research should focus on the improvement of exfoliation yield and structural stability, such as selecting a more efficient exfoliation solvent and/or surface modification on the nanosheets. Therefore, further studies and improvements in the exfoliation process are needed to propose basic principles and key factors which determine the exfoliation efficiency and nanosheet quality.

(iii) More attempts at superlattice assembly of rare earth-containing nanosheets and other functional nanosheets are expected. Molecular-scale assembly enables construction of functional nanostructures based on 2D rare earth nanomaterials with diverse compositions and structures, and is an important method to improve their properties, especially for rare earth-based or rare earth-containing nanosheets. It is proved that for superlattice-like assembly, the suitable pairing (combination) of rare earth-based or doped nanosheets with other functional nanosheets can significantly enhance the original properties (*e.g.*, photoluminescence properties) or even create new properties (*e.g.*, photothermal properties). Thus, more attempts on the superlattice assembly of rare earth-containing nanosheets and other functional nanosheets (*e.g.*, MXenes, TMDs, oxides, *etc.*) may spawn new interesting functional materials.

(iv) Expansion and acceleration on applications of 2D nanomaterials based on rare earth elements. 2D rare earth-based or doped nanomaterials are mainly applied to drug delivery and bioimaging, and have demonstrated excellent performance and promising application prospects in diverse biomedicine fields. Further investigations of rare earth nanomaterials in other frontier biomedical fields such as cancer treatment agents (*e.g.*, photothermal therapy, photodynamic therapy, *etc.*), biosensors, theragnostic agents, tissue engineering and regenerative agents are highly expected.

## Data availability

No primary research results, software or code has been included and no new data were generated or analysed as part of this review.

## Author contributions

M. B. and R. M. conceived the idea. H. W., X. L. and R. M. supervised the project. The manuscript was written through the contribution of all authors. All authors have approved the final version of the manuscript.

## Conflicts of interest

There are no conflicts to declare.

## References

[cit1] Novoselov K. S., Geim A. K., Morozov S. V., Jiang D., Zhang Y., Dubonos S. V., Grigorieva I. V., Firsov A. A. (2004). Science.

[cit2] Liu H., Neal A. T., Zhu Z., Luo Z., Xu X., Tománek D., Ye P. D. (2014). ACS Nano.

[cit3] Shi Z., Zhang Z., Kutana A., Yakobson B. I. (2015). ACS Nano.

[cit4] Watanabe K., Taniguchi T., Kanda H. (2004). Nat. Mater..

[cit5] Zhang Y., Pei Q., Wang C. (2012). Appl. Phys. Lett..

[cit6] Zhang W., Zhang X., Ono L. K., Qi Y., Oughaddou H. (2024). Small.

[cit7] Feng B., Zhang J., Zhong Q., Li W., Li S., Li H., Cheng P., Meng S., Chen L., Wu K. (2016). Nat. Chem..

[cit8] Mannix A. J., Zhou X.-F., Kiraly B., Wood J. D., Alducin D., Myers B. D., Liu X., Fisher B. L., Santiago U., Guest J. R. (2015). Science.

[cit9] Wang T., Wang H., Kou Z., Liang W., Luo X., Verpoort F., Zeng Y. J., Zhang H. (2020). Adv. Funct. Mater..

[cit10] Shan G., Tan H., Ma R., Zhao H. B., Huang W. (2023). Nanoscale.

[cit11] Duerloo K.-A. N., Li Y., Reed E. J. (2014). Nat. Commun..

[cit12] Lopez-Sanchez O., Lembke D., Kayci M., Radenovic A., Kis A. (2013). Nat. Nanotechnol..

[cit13] Sun C., Wang L., Zhao W., Xie L., Wang J., Li J., Li B., Liu S., Zhuang Z., Zhao Q. (2022). Adv. Funct. Mater..

[cit14] Kumar J. A., Prakash P., Krithiga T., Amarnath D. J., Premkumar J., Rajamohan N., Vasseghian Y., Saravanan P., Rajasimman M. (2022). Chemosphere.

[cit15] Naguib M., Barsoum M. W., Gogotsi Y. (2021). Adv. Mater..

[cit16] Lim K. R. G., Shekhirev M., Wyatt B. C., Anasori B., Gogotsi Y., Seh Z. W. (2022). Nat. Synth..

[cit17] Lin Y., Wan H., Wu D., Chen G., Zhang N., Liu X., Li J., Cao Y., Qiu G., Ma R. (2020). J. Am. Chem. Soc..

[cit18] Dong P., Gu Y., Wen G., Luo R., Bao S., Ma J., Lei J. (2023). Small.

[cit19] Knebel A. A., Caro J. (2022). Nat. Nanotechnol..

[cit20] Daliran S., Oveisi A. R., Peng Y., López-Magano A., Khajeh M., Mas-Ballesté R., Alemán J., Luque R., Garcia H. (2022). Chem. Soc. Rev..

[cit21] Ma S., Deng T., Li Z., Zhang Z., Jia J., Li Q., Wu G., Xia H., Yang S. W., Liu X. (2022). Angew Chem., Int. Ed..

[cit22] Haque F., Daeneke T., Kalantar-Zadeh K., Ou J. Z. (2018). Nano-Micro Lett..

[cit23] Kumar P., Liu J., Ranjan P., Hu Y., Yamijala S. S., Pati S. K., Irudayaraj J., Cheng G. J. (2018). Small.

[cit24] Stoumpos C. C., Cao D. H., Clark D. J., Young J., Rondinelli J. M., Jang J. I., Hupp J. T., Kanatzidis M. G. (2016). Chem. Mater..

[cit25] Bai M., Zhang Y., Wan H., Chen G., Liu X., Ma R. (2022). Chem. Commun..

[cit26] Xiong P., Ma R., Wang G., Sasaki T. (2019). Energy Storage Mater..

[cit27] Raadi Z., Rahimi A., Ghanbari H., Sarpoolaky H. (2022). Adv. Mater. Technol..

[cit28] Lu X., Xue H., Gong H., Bai M., Tang D., Ma R., Sasaki T. (2020). Nano-Micro Lett..

[cit29] Yi H., Liu S., Lai C., Zeng G., Li M., Liu X., Li B., Huo X., Qin L., Li L., Zhang M., Fu Y., An Z., Chen L. (2021). Adv. Energy Mater..

[cit30] Boumeriame H., Da Silva E. S., Cherevan A. S., Chafik T., Faria J. L., Eder D. (2022). J. Energy Chem..

[cit31] Liang J., Ma R., Sasaki T. (2023). Trends Chem..

[cit32] Zhu Q., Wang X., Li J.-G. (2017). J. Adv. Ceram..

[cit33] Bai M., Liu X., Sakai N., Ebina Y., Jia L., Tang D., Sasaki T., Ma R. (2021). J. Phys. Chem. Lett..

[cit34] Bai M., Li J., Liu X., Sasaki T., Ma R. (2022). Adv. Opt. Mater..

[cit35] Yu J., Xu Y., Shi S., Wang J., Song H., Fu L. (2022). Spectrochim. Acta, Part A.

[cit36] Jeon H. G., Kim H., Byeon S. H. (2019). Adv. Mater. Interfaces.

[cit37] Jeon H.-G., Kim H., Byeon S.-H. (2021). Chem. Eng. J..

[cit38] Zeng Z., Xu Y., Zhang Z., Gao Z., Luo M., Yin Z., Zhang C., Xu J., Huang B., Luo F. (2020). Chem. Soc. Rev..

[cit39] Wang X., Chen W., Song Y. F. (2014). Eur. J. Inorg. Chem..

[cit40] Xu J., Zhu K., Gao S., Hou Y. (2021). Inorg. Chem. Front..

[cit41] Lee B. I., Lee K. S., Lee J. H., Lee I. S., Byeon S. H. (2009). Dalton Trans..

[cit42] Yoon Y. s., Lee B. I., Lee K. S., Im G. H., Byeon S. H., Lee J. H., Lee I. S. (2009). Adv. Funct. Mater..

[cit43] Li X., Xue Z., Xia J., Zhou G., Jiang D., Dai M., Wang W., Miu J., Heng Y., Yu C. (2020). Nanomaterials.

[cit44] Li J., Duan J., He Z., Liao Y., Liu X., Rong P., Chen G., Wan H., Huang Y., Ma R. (2023). Adv. Opt. Mater..

[cit45] Gu Q., Li J., Ji L., Ju R., Jin H., Zhang R. (2020). Front. Mater. Sci..

[cit46] Strimaite M., Harman C. L. G., Duan H., Wang Y., Davies G. L., Williams G. R. (2021). Dalton Trans..

[cit47] Pinho S. L., Amaral J. S., Wattiaux A., Duttine M., Delville M. H., Geraldes C. F. (2018). Eur. J. Inorg. Chem..

[cit48] Aksel'rud N. (1963). Russ. Chem. Rev..

[cit49] Zhong Y., Chen G., Liu X., Zhang D., Zhang N., Li J., Liang S., Ma R., Qiu G. (2017). Nanoscale.

[cit50] Xiao Y., Chen G., Liu X., Bai M., Zhang N., Ma W., Ma R. (2018). CrystEngComm.

[cit51] Li J., Li J.-G., Zhu Q., Sun X. (2016). Mater. Des..

[cit52] Wu M., Li L., Yu X., Zhang D., Sun T., Li X., Sun L., Lui S., Huang X., Bi F. (2014). J. Biomed. Nanotechnol..

[cit53] Li J., Duan J., Liao Y., Liu X., Rong P., Chen G., Wan H., Ma R. (2023). Mater. Des..

[cit54] Gu Z., Wu A., Li L., Xu Z. P. (2014). Pharmaceutics.

[cit55] Bian Y., Cai X., Lv Z., Xu Y., Wang H., Tan C., Liang R., Weng X. (2023). Adv. Sci..

[cit56] Shirin V. A., Sankar R., Johnson A. P., Gangadharappa H., Pramod K. (2021). J. Controlled Release.

[cit57] Xu Y., Goyanes A., Wang Y., Weston A. J., So P.-W., Geraldes C. F., Fogg A. M., Basit A. W., Williams G. R. (2018). Dalton Trans..

[cit58] Ju R., Gu Q. (2018). Appl. Organomet. Chem..

[cit59] Lee B. I., Lee E. s., Byeon S. H. (2012). Adv. Funct. Mater..

[cit60] Yoon Y. S., Byeon S. H., Lee I. S. (2010). Adv. Mater..

[cit61] Bai M., Liu X., Sasaki T., Ma R. (2021). Nanoscale.

[cit62] Wang Y., Chen J., Duan R., Gu R., Wang W., Wu J., Lian H., Hu Y., Yuan A. (2022). Adv. Mater..

[cit63] Milstein T. J., Kluherz K. T., Kroupa D. M., Erickson C. S., De Yoreo J. J., Gamelin D. R. (2019). Nano Lett..

[cit64] Annadi A., Cheng G., Lee H., Lee J.-W., Lu S., Tylan-Tyler A., Briggeman M., Tomczyk M., Huang M., Pekker D. (2018). Nano Lett..

[cit65] Ida S., Ogata C., Eguchi M., Youngblood W. J., Mallouk T. E., Matsumoto Y. (2008). J. Am. Chem. Soc..

[cit66] Ozawa T. C., Fukuda K., Akatsuka K., Ebina Y., Sasaki T., Kurashima K., Kosuda K. (2008). J. Phys. Chem. C.

[cit67] Ozawa T. C., Fukuda K., Akatsuka K., Ebina Y., Sasaki T. (2007). Chem. Mater..

[cit68] Ozawa T. C., Fukuda K., Akatsuka K., Ebina Y., Sasaki T., Kurashima K., Kosuda K. (2008). J. Phys. Chem. C.

[cit69] Viswanath N., Arunkumar P., Kim H. J., Im W. B. (2019). Chem. Eng. J..

[cit70] Günay B., Döğer H., Karagonlar Z. F., Sağlam Ö. (2022). Mater. Today Commun..

[cit71] Shiroma Y., Mogi H., Mashiko T., Yasuda S., Nishioka S., Yokoi T., Ida S., Kimoto K., Maeda K. (2023). J. Mater. Chem. A.

[cit72] Panda D. P., Singh A. K., Kundu T. K., Sundaresan A. (2022). J. Mater. Chem. B.

[cit73] Yapryntsev A. D., Baranchikov A. E., Ivanov V. K. (2020). Russ. Chem. Rev..

[cit74] Hossain M. K., Hossain S., Ahmed M. H., Khan M. I., Haque N., Raihan G. A. (2021). ACS Appl. Electron. Mater..

[cit75] Hossain M. K., Ahmed M. H., Khan M. I., Miah M. S., Hossain S. (2021). ACS Appl. Electron. Mater..

[cit76] Chen F., Tang Q., Ma T., Zhu B., Wang L., He C., Luo X., Cao S., Ma L., Cheng C. (2022). InfoMat.

[cit77] Li Q., Wu X., Mu S., He C., Ren X., Luo X., Adeli M., Han X., Ma L., Cheng C. (2023). Adv. Sci..

[cit78] Chen P., Han W., Zhao M., Su J., Li Z., Li D., Pi L., Zhou X., Zhai T. (2021). Adv. Funct. Mater..

[cit79] Liu S., Zhao Y., Cao S., Chen S., Wang C., Shi X., Zhao H. (2024). Appl. Surf. Sci..

[cit80] Sokolov I. S., Averyanov D. V., Parfenov O. E., Karateev I. A., Taldenkov A. N., Tokmachev A. M., Storchak V. G. (2020). Mater. Horiz..

[cit81] Tokmachev A. M., Averyanov D. V., Parfenov O. E., Taldenkov A. N., Karateev I. A., Sokolov I. S., Kondratev O. A., Storchak V. G. (2018). Nat. Commun..

[cit82] Liu X., Zhao H., Chen Y., Liang X., Liu S., Huang Z., Wu Z., Mao Y., Shi X. (2024). Mater. Today Chem..

[cit83] Gándara F., Perles J., Snejko N., Iglesias M., GómezLor B., GutiérrezPuebla E., Monge M. Á. (2006). Angew Chem., Int. Ed..

[cit84] Newman S. P., Jones W. (1999). J. Solid State Chem..

[cit85] Geng F., Matsushita Y., Ma R., Xin H., Tanaka M., Izumi F., Iyi N., Sasaki T. (2008). J. Am. Chem. Soc..

[cit86] Poudret L., Prior T. J., McIntyre L. J., Fogg A. M. (2008). Chem. Mater..

[cit87] Liang J., Ma R., Geng F., Ebina Y., Sasaki T. (2010). Chem. Mater..

[cit88] Geng F., Xin H., Matsushita Y., Ma R., Tanaka M., Izumi F., Iyi N., Sasaki T. (2008). Chem.–Eur. J..

[cit89] Geng F., Matsushita Y., Ma R., Xin H., Tanaka M., Iyi N., Sasaki T. (2009). Inorg. Chem..

[cit90] Zhu Q., Li J.-G., Ma R., Sasaki T., Yang X., Li X., Sun X., Sakka Y. (2012). J. Solid State Chem..

[cit91] Geng F., Ma R., Sasaki T. (2010). Acc. Chem. Res..

[cit92] Liang J., Ma R., Sasaki T. (2014). Dalton Trans..

[cit93] Southworth F. Y., Wilson C., Coles S. J., Fogg A. M. (2014). Dalton Trans..

[cit94] Zhu Q., Xu Z., Li J. G., Li X., Qi Y., Sun X. (2015). Nanoscale Res. Lett..

[cit95] Xiao Y., Zhong Y., Liu X., Zhang N., Liang S., Ma R. (2018). Part. Part. Syst. Charact..

[cit96] Wang Z., Li J. G., Zhu Q., Li X., Sun X. (2016). Dalton Trans..

[cit97] Lee K. H., Byeon S. H. (2009). Eur. J. Inorg. Chem..

[cit98] Lee K. H., Byeon S. H. (2009). Eur. J. Inorg. Chem..

[cit99] Wu X., Li J.-G., Zhu Q., Liu W., Li J., Li X., Sun X., Sakka Y. (2015). J. Mater. Chem. C.

[cit100] Kim H., Lee B. I., Jeong H., Byeon S. H. (2015). J. Mater. Chem. C.

[cit101] Kim H., Lee B. I., Byeon S. H. (2015). Chem. Commun..

[cit102] Lee B. I., Bae J. S., Lee E. S., Byeon S. H. (2012). Bull. Korean Chem. Soc..

[cit103] Sokolov M. R., Enakieva Y. Y., Yapryntsev A. D., Shiryaev A. A., Zvyagina A. I., Kalinina M. A. (2020). Adv. Funct. Mater..

[cit104] Zhu Q., Li S., Jin J., Xu Z., Li X., Sun X., Li J. G. (2018). Chem.–Asian J..

[cit105] Zhu Q., Li S., Wang Q., Qi Y., Li X., Sun X., Li J.-G. (2019). Nanoscale.

[cit106] Gu Q., Chen W., Duan F., Ju R. (2016). Dalton Trans..

[cit107] Stefanakis D., Ghanotakis D. F. (2010). J. Nanopart. Res..

[cit108] Yoo S. S., Razzak R., Bédard E., Guo L., Shaw A. R., Moore R. B., Roa W. H. (2014). Nanotechnology.

[cit109] Hu L., Ma R., Ozawa T. C., Sasaki T. (2010). Chem.–Asian J..

[cit110] Li J.-G., Li J., Wang X., Zhu Q., Li X., Kim B.-N., Sun X. (2017). Dalton Trans..

[cit111] Lee K. H., Lee B. I., You J. H., Byeon S. H. (2010). Chem. Commun..

[cit112] Yapryntsev A. D., Ustinovich K. B., Rodina A. A., Lebedev V. A., Pokrovskiy O. I., Yorov K. E., Gavrikov A. V., Baranchikov A. E., Ivanov V. K. (2019). J. Supercrit. Fluids.

[cit113] Rodina A., Yapryntsev A., Churakov A., Baranchikov A. (2021). Russ. J. Inorg. Chem..

[cit114] Li J., Li M., Zhang Z., Zheng Z., Chen G., Wan H., Zhang Y., Liu X., Ma R. (2021). ACS Appl. Nano Mater..

[cit115] Maeno Y., Hashimoto H., Yoshida K., Nishizaki S., Fujita T., Bednorz J. G., Lichtenberg F. (1994). Nature.

[cit116] Wang T., Henderson C. N., Draskovic T. I., Mallouk T. E. (2014). Chem. Mater..

[cit117] Oshima T., Ichibha T., Oqmhula K., Hibino K., Mogi H., Yamashita S., Fujii K., Miseki Y., Hongo K., Lu D., Maezono R., Sayama K., Yashima M., Kimoto K., Kato H., Kakihana M., Kageyama H., Maeda K. (2020). Angew Chem., Int. Ed..

[cit118] Machida M., Mitsuyama T., Ikeue K., Matsushima S., Arai M. (2005). J. Phys. Chem. B.

[cit119] Machida M., Miyazaki K., Matsushima S., Arai M. (2003). J. Mater. Chem..

[cit120] Deng B., Jiang J., Chen W., Zhang A., Liang Z., Li F., Zeng F., Zhang G. (2022). Inorg. Chem. Commun..

[cit121] Schaak R. E., Mallouk T. E. (2000). Chem. Mater..

[cit122] Gunay B., Sarıyar E., Unal U., Karagonlar Z. F., Sağlam Ö. (2021). Colloids Surf., A.

[cit123] Fu L.-M., Lin B.-Z., Chan Y.-L., Zhang O., Li B., Qu H. (2012). J. Alloys Compd..

[cit124] Sağlam Ö. (2020). Opt. Mater..

[cit125] Gunay B., Süer Ö., Döğer H., Arslan Ö., Unal U., Sağlam Ö. (2022). Colloids Surf., A.

[cit126] Sharits A. R., Khoury J. F., Woodward P. M. (2016). Inorg. Chem..

[cit127] Meng S., Li G., Wang P., He M., Sun X., Li Z. (2023). Mater. Chem. Front..

[cit128] Liu Q., Li X. F., Wen Y. H., Xu Q. D., Wu X. T., Zhu Q. L. (2020). Adv. Mater. Interfaces.

[cit129] Wang X. Y., Xiao S. Y., Jiang Z. W., Zhen S. J., Huang C. Z., Liu Q. Q., Li Y. F. (2021). Talanta.

[cit130] Jiang Z. W., Zou Y. C., Zhao T. T., Zhen S. J., Li Y. F., Huang C.
Z. (2020). Angew Chem. Int. Ed. Engl..

[cit131] Jiang Z. W., Zhao T. T., Zhen S. J., Li C. M., Li Y. F., Huang C. Z. (2021). J. Mater. Chem. A.

[cit132] Wang H.-S., Li J., Li J.-Y., Wang K., Ding Y., Xia X.-H. (2017). NPG Asia Mater..

[cit133] Wang X., Jiang Z., Yang C., Zhen S., Huang C., Li Y. (2022). J. Hazard. Mater..

[cit134] Huang W., Pan F., Liu Y., Huang S., Li Y., Yong J., Li Y., Kirillov A. M., Wu D. (2017). Inorg. Chem..

[cit135] Tian C., Pan F., Wang L., Ye D., Sheng J., Wang J., Liu J., Huang J., Zhang H., Xu D. (2021). Phys. Rev. B.

[cit136] Chen P., Li Z., Li D., Pi L., Liu X., Luo J., Zhou X., Zhai T. (2021). Small.

[cit137] Zhang B., Zhu Y., Zeng Y., Zhao Z., Huang X., Qiu D., Fang Z., Wang J., Xu J., Wang R. (2023). J. Am. Chem. Soc..

[cit138] Hu L., Ma R., Ozawa T. C., Sasaki T. (2009). Angew Chem., Int. Ed..

[cit139] Hu L., Ma R., Ozawa T. C., Geng F., Iyi N., Sasaki T. (2008). Chem. Commun..

[cit140] Huang J., Zhang T., Ren K., Zhang R., Wu X., Li J.-g. (2018). J. Alloys Compd..

[cit141] Ida S., Sonoda Y., Ikeue K., Matsumoto Y. (2010). Chem. Commun..

[cit142] Lee B. I., Jeong H., Byeon S. H. (2014). Eur. J. Inorg. Chem..

[cit143] Xin H., Ma R., Wang L., Ebina Y., Takada K., Sasaki T. (2004). Appl. Phys. Lett..

[cit144] Ozawa T. C., Onoda M., Iyi N., Ebina Y., Sasaki T. (2014). J. Phys. Chem. C.

[cit145] Zhang N., Li C., Jiang D., Chu J., Chen H., Zhang J., Li Q. (2010). J. Alloys Compd..

[cit146] Bai M., Sutrisno L., Duan J., Wan H., Chen G., Liu X., Ma R. (2023). Chem. Sci..

[cit147] Xia J., Xue Y., Lei B., Xu L., Sun M., Li N., Zhao H., Wang M., Luo M., Zhang C., Huang B., Du Y., Yan C.-H. (2020). Natl. Sci. Rev..

[cit148] Mei X., Ma J., Bai X., Zhang X., Zhang S., Liang R., Wei M., Evans D. G., Duan X. (2018). Chem. Sci..

[cit149] Nava Andrade K., Knauth P., López Z., Hirata G. A., Guevara Martinez S. J., Carbajal Arízaga G. G. (2020). Appl. Clay Sci..

[cit150] Liu J., Li L., Zhang R., Xu Z. P. (2023). Nanoscale Horiz..

[cit151] Xu Z. P., Kurniawan N. D., Bartlett P. F., Lu G. Q. (2007). Chem.–Eur. J..

[cit152] Jung S.-Y., Park J. K., Oh J.-M. (2021). Clays Clay Miner..

[cit153] Wang L., Zhang B., Yang X., Guo S., Waterhouse G. I. N., Song G., Guan S., Liu A., Cheng L., Zhou S. (2023). Bioact. Mater..

[cit154] Jia T., Wang Z., Sun Q., Dong S., Xu J., Zhang F., Feng L., He F., Yang D., Yang P., Lin J. (2020). Small.

[cit155] Guan S., Liang R., Li C., Wei M. (2017). Talanta.

[cit156] Mei X., Wang W., Yan L., Hu T., Liang R., Yan D., Wei M., Evans D. G., Duan X. (2018). Biomaterials.

[cit157] Jung S.-Y., Gwak G.-H., Park J. K., Oh J.-M. (2020). RSC Adv..

[cit158] Wang Z., Fu L., Zhu Y., Wang S., Shen G., Jin L., Liang R. (2021). J. Mater. Chem. B.

[cit159] Zhang R., Chen G., Du J., Wang Q., Qi Q., Li X., Zhu L., Chen X., Liu B., Miao Y., Li Y. (2023). Chem. Eng. J..

[cit160] Du J., He Z., Wang Q., Chen G., Li X., Lu J., Qi Q., Ouyang R., Miao Y., Li Y. (2024). J. Colloid Interface Sci..

[cit161] Yang Y., Hu T., Bian Y., Meng F., Yu S., Li H., Zhang Q., Gu L., Weng X., Tan C., Liang R. (2023). Adv. Mater..

[cit162] Peng L., Mei X., He J., Xu J., Zhang W., Liang R., Wei M., Evans D. G., Duan X. (2018). Adv. Mater..

[cit163] Zheng B., Fan J., Chen B., Qin X., Wang J., Wang F., Deng R., Liu X. (2022). Chem. Rev..

[cit164] Ramu S., Vijayalakshmi R. (2017). J. Supercond. Novel Magn..

[cit165] Ruan M., Ouyang Z., Wang Z., Xia Z., Rao G. (2017). Appl. Phys. Lett..

[cit166] Almessiere M. A., Slimani Y., Güngüneş H., Kostishyn V. G., Trukhanov S. V., Trukhanov A. V., Baykal A. (2020). Ceram. Int..

[cit167] Wan H., Rong P., Liu X., Yang L., Jiang Y., Zhang N., Ma R., Liang S., Wang H., Qiu G. (2017). Adv. Funct. Mater..

[cit168] Ma K., Chen K.-Z., Qiao S.-L. (2024). Chem. Rec..

[cit169] Cui H., Zhao Y.-Y., Wu Q., You Y., Lan Z., Zou K.-L., Cheng G.-W., Chen H., Han Y.-H., Chen Y., Qi X.-D., Meng X.-W., Ma L.-M., Yu G.-T. (2024). Bioact. Mater..

[cit170] Jia M., Yang X., Chen Y., He M., Zhou W., Lin J., An L., Yang S. (2021). J. Mater. Chem. B.

[cit171] Liu H., Li J., Hu P., Sun S., Shi L., Sun L. (2022). J. Rare Earths.

[cit172] Xu J., Shi R., Chen G., Dong S., Yang P., Zhang Z., Niu N., Gai S., He F., Fu Y., Lin J. (2020). ACS Nano.

[cit173] Liu Y., Xu Y., Geng X., Huo Y., Chen D., Sun K., Zhou G., Chen B., Tao K. (2018). Small.

